# Understanding barriers and facilitators to education and rehabilitation interventions for South Asian people with long-term conditions: a systematic review and meta-ethnography

**DOI:** 10.1136/bmjopen-2025-106694

**Published:** 2026-01-13

**Authors:** Emma Victoria Shiel, Jahanara Miah, Tapan Chattopadhyay, Abdur Rauf, Christopher Dalton, Nusrat Husain, Amy Blakemore

**Affiliations:** 1 Division of Nursing, Midwifery and Social Work, School of Health Sciences, Faculty of Biology Medicine and Health, University of Manchester, Manchester, UK; 2 Division of Psychology and Mental Health, School of Health Sciences, Faculty of Biology, Medicine and Health, University of Manchester, Manchester, UK; 3 Expert by Experience, Manchester, UK; 4 Ethnic Health Forum, Manchester, UK; 5 Wrightingtion, Wigan and Leigh NHS Foundation Trust, Royal Albert Edward Infirmary, Wigan, UK; 6 Mersey Care NHS Foundation Trust, Prescot, Merseyside, UK

**Keywords:** Coronary heart disease, Pulmonary Disease, Chronic Obstructive, DIABETES & ENDOCRINOLOGY, REHABILITATION MEDICINE, Patient Participation

## Abstract

**Abstract:**

**Objectives:**

South Asian populations in the UK experience increased health risks related to long-term conditions, exacerbated by underdiagnosis, cultural differences in help-seeking behaviours, language barriers, low health literacy and a lack of culturally sensitive services. We know that group interventions that include education and rehabilitation, such as cardiac and pulmonary rehabilitation, are highly effective, but people from diverse communities often face barriers to access and engage with them. This review aims to synthesise evidence on the barriers and facilitators to education and rehabilitation interventions experienced by South Asian people living with long-term conditions.

**Design:**

A systematic review of qualitative studies using meta-ethnography as the analytical approach to synthesis was conducted, following Noblit and Hare’s approach, eMERGe Reporting Guidance for Meta-Ethnography, and Preferred Reporting Items for Systematic Reviews and Meta-Analyses (PRISMA) guidelines. Systematic searches were performed across MEDLINE, PsycINFO, CINAHL, CENTRAL, EMBASE and Applied Social Sciences Index and Abstracts from database inception through March 2024 (updated April 2025).

**Data sources:**

MEDLINE (Ovid), PsycINFO (Ovid), CINAHL (EBSCOhost platform), CENTRAL (Cochrane Library), EMBASE (Ovid), Applied Social Sciences Index and Abstracts (ProQuest platform) were searched from inception to March 2024 (updated April 2025).

**Eligibility criteria:**

We included qualitative research exploring the attitudes, views and experiences of South Asian adults (outside of South Asia) with diabetes, cardiovascular disease or chronic obstructive pulmonary disease (COPD) regarding group treatments for these conditions.

**Data extraction and synthesis:**

Two independent reviewers searched, screened and coded studies, while remaining authors peer-reviewed. Study quality was assessed using the Joanna Briggs Institute Critical Appraisal Checklist for Qualitative Research. Data extraction and synthesis followed eMERGe and PRISMA reporting guidance, with findings synthesised qualitatively.

**Results:**

Of 8348 identified citations, 17 studies met inclusion criteria, providing data from South Asian people living with cardiovascular disease and diabetes mellitus. No studies including people with COPD met the inclusion criteria. Synthesis revealed four overarching themes, each incorporating both barriers and facilitators: faith, culture, communication, and safe space and professional relationship.

**Conclusions:**

Findings indicate that current group education and rehabilitation interventions are not fully inclusive of South Asian needs, often lacking cultural sensitivity, which impedes engagement. Special attention is required for South Asian women, who can face additional cultural and societal barriers. Addressing these challenges through culturally sensitive care, such as flexible intervention scheduling around religious practices, gender-sensitive adaptations and culturally tailored communication strategies, has potential to improve engagement in education and rehabilitation interventions, and therefore long-term condition outcomes.

**PROSPERO registration number:**

CRD42024493644.

**STRENGTHS AND LIMITATIONS OF THIS STUDY**

- The search strategy was validated, and protocol registered prospectively.
- The meta-ethnography followed Noblit and Hare’s approach with both reciprocal translation and line-of-argument synthesis.
- The use of rigorous and explorative meta-ethnography methods meant new theory was developed.
- The searches were systematic, comprehensive, conducted across six databases and supplemented with citation searching.
- This synthesis did not include non-peer reviewed articles, so it may be affected by publication bias.

## Background

 The South Asian population in the UK has grown significantly, now comprising 6.9% of the total population.^1 2^ This population faces disproportionate health challenges, with twofold higher incidence of coronary heart disease (CHD) and two to six times increased risk of type 2 diabetes mellitus (T2DM) compared with white Europeans.^3 4^ While the rate of chronic obstructive pulmonary disease (COPD) is lower, this likely reflects significant underdiagnosis due to reduced help-seeking, cultural health beliefs and limited lung health awareness in South Asian communities.^5^

Group education and rehabilitation interventions like cardiac rehabilitation (CR) and pulmonary rehabilitation (PR) represent gold-standard treatments for these conditions.^6 7^ Diabetes mellitus education programmes are also good practice. However, access and engagement rates for South Asian people living with long-term conditions (LTCs) are often low.^8^,^10^ Cultural explanatory models of illness in the South Asian community may affect engagement with these services,^11^ as people might not seek out medical help or follow traditional treatments. Cultural beliefs, particularly beliefs that LTCs are fate, can act as a barrier to help-seeking.^12^ So, South Asian people may not perceive the benefits from group rehabilitation programmes, nor view them as essential.^13 14^

Practical barriers include lack of translated materials, health literacy, lack of culturally sensitive services and transportation difficulties.^15 16^ When interventions are appropriately tailored for South Asian people, access, engagement and outcomes may improve.^17^ Adaptations regarding access, programme structure, language and communication approaches may be useful.^14 18^

Limited access and engagement to effective education and rehabilitation interventions for South Asian people living with LTCs in the UK is an important health inequality. Lack of access for the South Asian community may impose substantial costs on the National Health Service, highlighting the importance of optimising treatment approaches.

Therefore, this systematic review and meta-ethnography aims to synthesise evidence on the experience of South Asian people when attending group treatment programmes for LTCs including diabetes, cardiovascular disease and COPD. We are particularly interested in understanding the views, opinions, attitudes and experiences of South Asian people; therefore, a meta-ethnographic approach was appropriate to ensure findings contribute to the growing body of qualitative literature on this topic.

## Methods

This systematic review follows Noblit and Hare’s^19^ meta-ethnography methodology, eMERGe Reporting Guidance for Meta-Ethnography^20^ (see online supplemental material S1), and Preferred Reporting Items for Systematic Reviews and Meta-Analyses (PRISMA) guidelines.^21^ The protocol was prospectively registered with PROSPERO (2024): CRD42024493644.

### Patient and public involvement

This review was conducted as part of a National Institute for Health Research (NIHR)-funded project in which people with lived experience of COPD were involved in the development of the research question and consulted on the design of the project. Our patient and public co-applicant (TC) contributed to designing and writing the systematic review, while our Lived Experience Advisory Group (LEAP) comprising three South Asian individuals with lived experience of LTCs provided ongoing input on how we interpreted and presented the data, especially regarding the line of argument synthesis. Importantly, the LEAP provided ongoing input throughout the review process, rather than a single consultation, and is expected to be involved in future dissemination events and grants.

### Analytic approach

Meta-ethnography was chosen as the most appropriate synthesis method because the study aimed to understand how South Asian people with LTCs experience and make sense of the factors that influence their access to and engagement with group interventions. This interpretive approach enables a deeper synthesis of qualitative studies, moving beyond simply identifying and listing barriers and facilitators to produce new, novel interpretations while preserving the original meanings and study concepts.^19 22 23^

The following seven steps were undertaken, which are iterative and tend to overlap.

### Step 1: getting started

We began by consulting with patients and professionals. This highlighted limited awareness of education and rehabilitation interventions in the South Asian community. Scoping the literature also revealed limited data and no previous meta-synthesis or meta-ethnography of qualitative literature on this topic. Using the SPIDER tool for qualitative evidence synthesis^24^ (see box 1), we formulated the question: ‘What is the experience of South Asian people with LTCs regarding the barriers and facilitators to accessing, engaging with, and completing of group interventions?’. This approach aims to reinterpret existing research, offering deeper insights specific to this population, for which a meta-ethnography is essential.^25^

Box 1 **Inclusion criteria**

**S**ample: South Asian adults (aged over 18 years) with LTCs, specifically diabetes mellitus (any type), cardiovascular disease and COPD, living outside of South Asia. Samples with comorbid long-term physical and mental health conditions will also be included where either COPD, diabetes mellitus or cardiovascular disease is the primary condition.

**P**henomenon of **I**nterest: The attitudes, views and experiences of South Asian people about group treatments for LTCs delivered in the community, primary care and secondary care.

**D**esign: Qualitative research methods, such as semi-structured interviews, focus groups and observational methods.

**E**valuation: We will synthesise data from articles that have explored the attitudes, views and experiences of South Asian people with diabetes mellitus, cardiovascular disease or COPD, around group interventions, and identified barriers and facilitators to access, engagement and completion of these groups.

**R**esearch type: Qualitative articles, such as those using semi-structured interviews, focus groups and observational methods.

COPD, chronic obstructive pulmonary disease; LTCs, long-term conditions.

### Step 2: deciding what is relevant

The following databases were systematically searched to retrieve peer-reviewed papers: MEDLINE (Ovid), PsycINFO (Ovid), CINAHL (EBSCOhost platform), CENTRAL (Cochrane Library), EMBASE (Ovid), Applied Social Sciences Index and Abstracts (ProQuest platform), from inception to March 2024 (updated April 2025).

Articles were selected based on predetermined inclusion criteria (see box 1) defined by the scope of the research question, and the specific requirements of meta-ethnography, that is, in-depth qualitative data rich in direct quotations. Search processes followed PRISMA guidelines.^21^

Inclusion criteria were intentionally broad to capture an array of relevant concepts until theoretical saturation was achieved.^20^ Articles that did not meet the inclusion criteria but contained pertinent data were reviewed, and only the relevant data was extracted for analysis. Grey literature and non-published articles were excluded. Searching was supplemented by citation searching and index tracking. We conducted systematic searches across six electronic databases from inception to March 2024 (updated April 2025): MEDLINE, PsycINFO and EMBASE (via Ovid), CINAHL (via EBSCOhost), CENTRAL (via Cochrane Library), and Applied Social Sciences Index and Abstracts (via ProQuest).

The search strategy comprised four key components combined using Boolean operators:

1. Condition terms for COPD, cardiovascular disease and diabetes.
2. Intervention terms, for example, self-management, patient education, health promotion, disease management and rehabilitation programmes.
3. Population terms for South Asian countries (ie, India, Pakistan, Bangladesh, Sri Lanka, Nepal, Bhutan, Afghanistan, Maldives) and South Asian ethnic groups.
4. Qualitative research methodology terms, for example, interviews, focus groups, phenomenology, ethnography.

Search terms were adapted for each database using appropriate Medical Subject Headings terms and free-text keywords. The full search strategy is provided inonline supplemental material S2.

**Figure 1:**
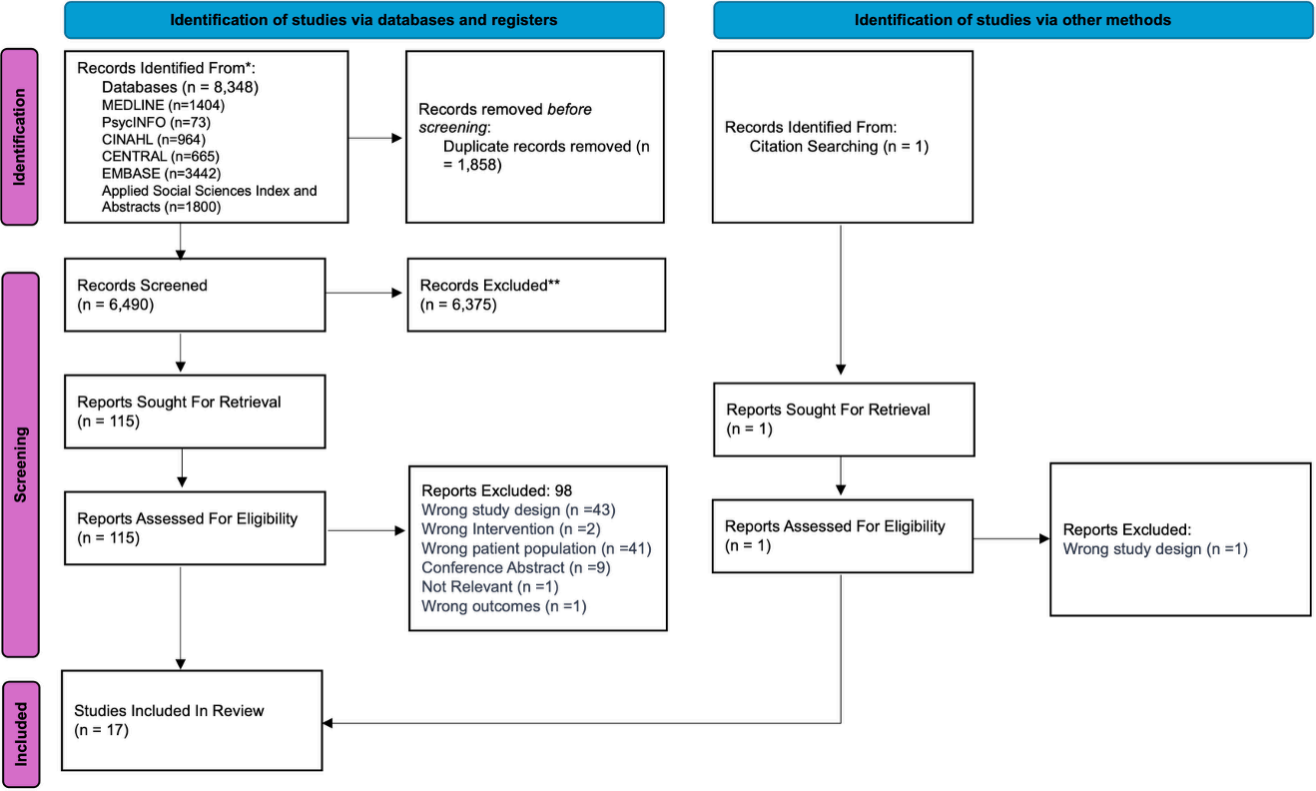
PRISMA (Preferred Reporting Items for Systematic Reviews and Meta-Analyses) flow diagram.

Articles were independently screened in Covidence (by title and abstract and then full text) by two reviewers (EVS and JM). Conflicts were resolved through discussion and approved by another author (AB). The review team brought complementary perspectives to study selection and interpretation: EVS, a qualitative health services researcher with lived experience of an LTC; JM, a specialist in community engagement and involvement in research; and AB, a health services researcher with expertise in quality of life in COPD and experience designing and evaluating mental health interventions for people with LTCs in South Asia. The team’s diverse professional and personal experiences informed how studies were read and understood, while regular discussions helped to surface and reflect on differing assumptions and interpretations during screening and data familiarisation. 8348 articles were identified, of which 17 were eligible for inclusion (see figure 1).

### Step 3: reading the studies

To remain close to the data, all articles were repeatedly read throughout by two authors (EVS and AB). EVS conducted open coding and extracted interpretive metaphors, which were noted to aid analysis.

### Data extraction

Data extraction was first piloted on five articles, including: study characteristics (author information, year, country, population, health condition, type of group treatment, data collection method, analysis, research question/aim and key findings), and first and second-order constructs, later developed into a table of concepts. These constructs were independently extracted by EVS and repeated for accuracy by AB. Authors of included studies were contacted when papers had missing data, required clarification or additional quotations.

### Step 4: determining how the studies are related

First and second-order constructs from each article were compared, forming narratives and informed codes that reflected the data. Codes were grouped into six sets, each containing different articles, relating to the barriers and facilitators of group interventions for South Asian people with LTCs: (1) Language and Communication, (2) Family and Social Support, (3) Cultural and Religious Factors, (4) Knowledge and Education, (5) Accessibility and (6) Medical and Professional Roles. Based on the similarities within the data, reciprocal synthesis was decided.

### Step 5: translating the studies into one another

All included articles were appraised for methodological and conceptual quality using the Joanna Briggs Institute Critical Appraisal Checklist for Qualitative Research^26^ by two independent reviewers (EVS and AB) and subsequently ranked from highest to lowest. Ranking did not determine inclusion but informed the interpretive weighting of each study during synthesis, ensuring that higher-quality studies had greater influence on emerging concepts, while insights from lower-quality studies were treated cautiously but not excluded.^19^ A summary of the appraisal is given in online supplemental material S3. Studies were then reciprocally translated^22 27^ (online supplemental material S4), with translation considered successful when each concept and metaphor both accurately represented the original data and contributed new interpretive insights. Key findings were preserved in diagram format, providing a transparent audit trail that informed the subsequent line of argument synthesis and supported the overall trustworthiness of this review.

### Step 6: synthesising translations

**Figure 2:**
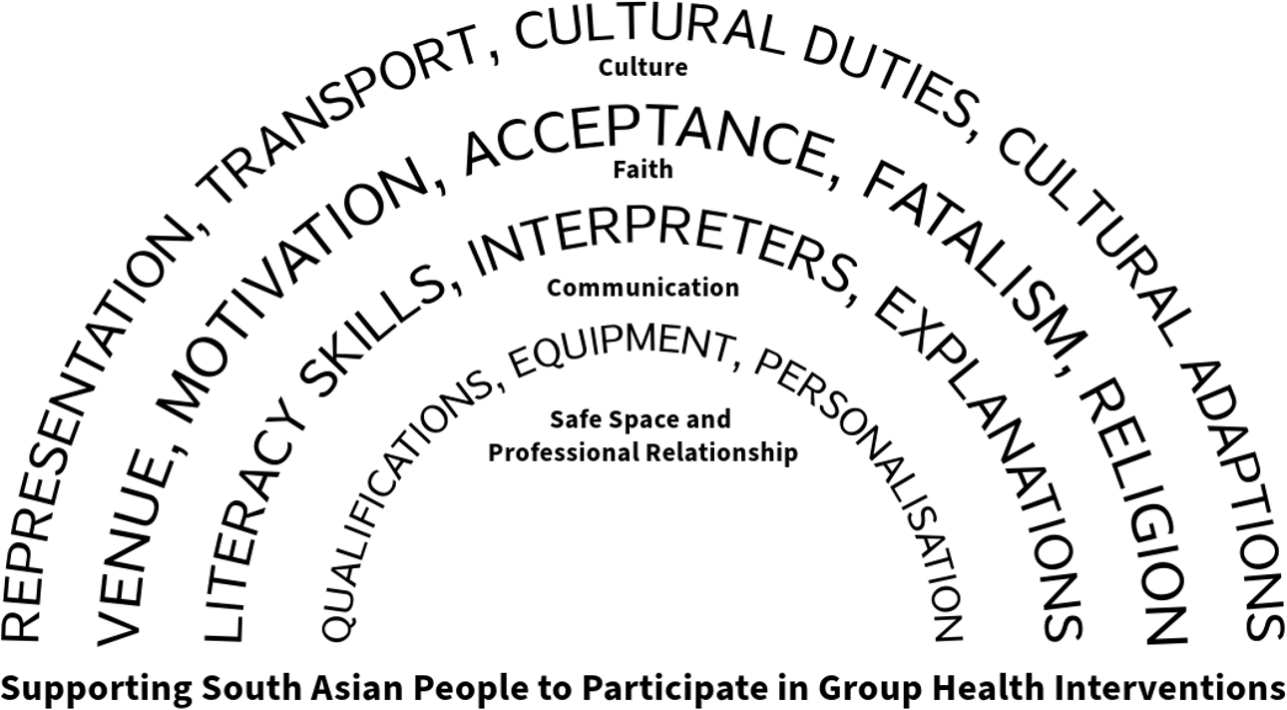
Line of argument synthesis.

A line of argument synthesis was conducted (figure 2). Articles were re-read to ensure the findings accurately represented the data set, grounding new insights in evidence, preserving their conceptual richness.^20^

Of the six translations in Step 5, four third-order constructs emerged: (1) Faith, (2) Culture, (3) Communication and (4) Safe space and professional relationship. The translations and articles included in each construct are provided (online supplemental material S5). Overall, 17 articles were translated; none were excluded.

During the translation and synthesis stages, EVS and AB engaged in regular reflexive discussions, both between themselves and with the LEAP to consider how their experiential perspectives influenced the interpretation of concepts and relationships across studies. Interpretations were then discussed with the remaining authors, including JM, a specialist in community engagement and involvement in research; TC, a retired geriatrician with lived experience of COPD; AR, Research, Strategy and Innovation Lead for a charity supporting minority ethnic communities to meet their health and social care needs; CD, a specialist physiotherapist with experience engaging South Asian people to access and engage with pulmonary rehabilitation; and NH with extensive experience in applied research with culturally adapted psychosocial and pharmacological treatments. Four authors (JM, TC, AR and NH) identify as British South Asian, representing Pakistan, Bangladesh and India, and providing additional cultural insight into the interpretation of data. Reflexive discussions throughout the synthesis process helped surface and manage potential biases. Our interpretations were also discussed with our LEAP (members described earlier) and Research Advisory Group, which included research and clinical professionals working in LTC healthcare and research settings. Differences in interpretation were explored collaboratively, documented by EVS and incorporated into the line-of-argument synthesis, which was subsequently refined further through consultation with the LEAP.

### Step 7: expressing the synthesis

Findings were expressed following the eMERGE Guidelines.^20^ Additional quotes are provided (online supplemental material S6). The presentation of the synthesis was informed by ongoing reflexive discussions between EVS and AB, with interpretations subsequently sense-checked with the full author team and LEAP to ensure the synthesis accurately reflected the data and considered multiple perspectives, including healthcare professionals (HCPs), researchers, community engagement and lived experience.

## Results

17 articles were synthesised; cardiovascular diseases (n=14),^1828^,^40^ T2DM (n=2)^41 42^ and gestational diabetes (n=1).^43^ While no eligible studies were identified that focused on COPD, insights are potentially transferable to programmes such as PR. Data fell into four themes: Culture, Faith, Communication and Safe Space and Professional Relationship. Full data extraction is given (see online supplemental material S7).

### Synthesis

#### Theme 1: Culture

Findings suggest that health is a personal responsibility, not a choice (n=4).

I don’t do it for myself I do it for them [family].^28^

But cultural norms and westernised advice hinder intervention participation (n=5). While living in larger multigenerational households could increase support, it made prioritising the needs of others difficult, as everyone must actively contribute.

I just tend to cook for the whole of the family and then just eat from that. I mean I could separate some for myself, but I don’t think there’s any point.^33^

As households generally adhered to male needs, females struggled to implement learning from interventions. So, women tended to their own needs separately, but this was not always possible.

Mixed-gender exercise was another obstacle for women who felt uncomfortable or were not permitted to attend such activities (n=2).

I think women won’t do exercises where men are around and to some its religious I think. It’s sinful to do exercises or the family might be saying you can’t go and do exercise, so I think it’s more cultural than anything else not to go and do exercises.^36^

Women rarely participated in physical activity, making the transition challenging, especially around others. Limited knowledge of gym equipment exacerbated worries.

The women say that the sessions were so new, and that they had never seen a place like this before, let alone done any exercise.^37^

Women-only groups were helpful but not a solution as there was hesitance to remove cultural garments for exercise. This could limit movement and make participation in exercise difficult, but sports attire often made people self-conscious or was not culturally appropriate.

I think [the patient] got the impression that because I had a tracksuit on ‘here is a wannabe English person’.^37^

Family support could be beneficial, but excessive family involvement can also raise barriers (n=2). Family members were often discouraged from strenuous exercise, and children who translated for their parents often concealed negative health news.

…if it’s like something bad that can happen, I don’t tell her I think she’s going to start thinking and then she’ll get worse, so we don’t tell her.^33^

Transport to and from group interventions also presented cultural challenges. While taxis were useful, many women lacked chaperones to use this service. Culturally inappropriate gestures like use of horns by the taxi driver also deterred use.

Cultural representation within the group setting emerged as a key facilitator (n=7), instilling belonging and comfort.

When you’re able to speak to someone that’s likeminded you’re able to have a dialogue and you’re able to share your experiences and they’re able to understand you and there’s a lot of support in that.^30^

Relatability among nursing staff built trust and made it easier to communicate interventions. However, providing such tailored treatment could be straining given the diversity within South Asian communities.

#### Theme 2: faith

Five articles identified faith as an obstacle to group interventions. Spiritual healing tended to be preferred over treatment.

If I prayer and prayer I can become better, that is also going in my mind… after all who knows what is there in the medicines? All is polluted, God only has the power to make clean my soul and body.^28^

One study examined the influence of deities such as ‘Bani,’ whose teachings suggested healthcare was unnecessary. Fatalistic attitudes further impeded participation.

You’re going to die when you are going to die, no one can stop that if… you listen to the doctor and you reduce your diet, you still have to die according to your time.^42^

Illness was commonly viewed as predestined (n=3). Thus, treatments were rendered futile. Instead, many learnt to cope and accept illness, vastly reducing the likelihood of seeking medical help. Especially as religious practices were prioritised over anything, including medical treatments.

Religion comes even before myself. [28]

Although excessive acceptance of their illness could sometimes deter participation, for some, it helped people to acknowledge their need for treatment and support, as well as take responsibility over their health.

God gave you good health. But I did not look after it. I ate what was not good for me and made it like this. But it must be written in my fate. But it’s my responsibility!.^28^

While spiritual beliefs can create barriers to interventions, they simultaneously offer frameworks that can introduce a positive perspective on sickness and help-seeking behaviours (n=2), such as maintaining independency and quality of life.

When a person becomes ill and sits around he becomes helpless, he is dependent… But if you get up and walk around, you get out and about, you are not dependent on anyone, that’s the only difference there’s nothing else you’re going to go when it’s your time.^42^

Religious venues further encouraged participation.

Yes—definitely a good place. The Gurdwara is a focal point in the lives of people health is part of that.^42^

The Gurdwara, associated with positive concepts such as health, shelter and equality, proved ideal for increasing accessibility and motivation to attend interventions. Familiarity with the venue was viewed to increase trust and comfort when attending.

#### Theme 3: communication

Generally, communication towards South Asian patients is poor (n=12). Systemic issues, particularly those related to language, hinder South Asian patients from visiting their general practitioners (GPs).

You see because of the language barrier and because how the system works here, our people find it difficult to go to the GPs.^42^

This meant many people had poor understanding of their health condition, treatments and group interventions. Many patients were unaware of rehabilitation programmes or the role of nurses. Some received information but did not understand its purpose. While several people knew English, their language and literacy skills were still limited.

…we have a bit of a problem with English. In Punjabi, we can ask something in full. We can ask questions in full, what is this, what is that, what isn’t it. In English, we don’t always understand everything.^30^

Atypical presentations of symptoms exacerbated communication issues as describing symptoms became increasingly difficult. Time constraints left patients feeling rushed, with insufficient time to fully understand diagnoses and recovery. This led to increased misunderstandings and missed appointments, resulting in shared losses for both patients and HCPs. Poor access to interventions further contributed to this (n=3).

Some HCPs assumed patients did not speak English, leading to poor engagement and a perceived lack of self-esteem and confidence in speaking with HCPs. Other papers showed physical health to also limit means of communication (n=2).

Poor literacy skills meant provided materials were inaccessible (n=3), or overwhelming.

They [CR nurses] gave me a cassette [about CR] and said put it on and read this. I cannot read English or understand it.^36^

Seeing images and facial expressions was crucial for non-English speaking patients to confirm their understanding. Visual cues, like DVDs and people’s emotions (eg, happy or sad faces), facilitated communication across languages and cultures. This simplicity was vital for many patients to attend.

DVD much better because it shows you in front of you, yeah, there is something picture wise showing instead of just book wise, you know, anyone telling you verbally or whatever, yeah.^41^

Interpreters were often used—when resources allowed—but interpreters were not always helpful.

The person who’s doing the talk gets put off because somebody else is talking and the other people are sitting round. It’s awkward for them as well because they are listening to us and trying to listen to what the interpreter is trying to say.^37^

English-speaking children helped; but led to exclusion.

They used to talk to my son and ask him any questions because I didn’t speak any English. So, they didn’t ask me anything or told me anything.^36^

Multi-lingual staff were uncommon but effective (n=2).

Lack of cultural sensitivity and knowledge was common, leading to unhelpful assumptions and biases (n=7).

The ambulance crew were talking amongst themselves about heart attack, and I understand English well. They didn’t tell me…I could not ask them anything because I had difficulty breathing and could not talk.^36^

These biases led patients to feel interventions, or even seeing the doctor, would be unhelpful.

Why I go? That’s the question. I talk to my doctor, he said, ‘Doesn’t need, up to you.’ So, I just control myself. I don’t eat much. I have good health. I do exercise, I joined the club here.^34^

As a result, mistrust may develop, decreasing the likelihood of South Asian people engaging with healthcare and interventions due to feeling unsafe or uncared for.

No, I did not tell the doctors. They wouldn’t understand. See… What do they know? Being in the state of purification for prayer? No. first they do not have time, second they will not understand.^28^

#### Theme 4: safe space and professional relationship

Feeling safe was integral to retention (n=6).

There’s the girl [at CR] that checks your ‘blood-pressure’, or the one that tells about exercise, there’s a big difference from them, because a person can see where his blood pressure has stabilized, or my pulse has stabilized there, and I should only do that much exercise, I shouldn’t do anymore. If I wanted to do more then I could ask them so that there is no danger from it.^18^

Being monitored by the nursing team provided safety, helping patients understand their limits—especially during exercise. This personalisation was important to participants as it empowered them to exercise alone and more frequently, improving their physical and psychological well-being. It also improved the relationship between patients and HCPs, which increased interest in interventions.

I really like the personal touch of the different people here. Obviously they call you by your first name which makes a little bit of a difference. They genuinely seem like they care, and to me that means a lot.^35^

As HCPs are trusted and respected in South Asian culture, participants usually attended if held responsible by their doctor. Qualifications and proof of expertise also encouraged participation.

…they still want medical staff with them in case they get chest pains… they feel more comfortable when [the Nurse] is here.^37^

Collaborative relationships helped patients feel pride and empowerment.

We can tell them [the CR healthcare professionals] what we’re doing, but afterwards we can phone them and say this is what we did because of the lessons that you’ve taught us. This is how much I’ve decreased my waist measure. This is how much my cholesterol has gone down.^30^

Reflecting on their successes with HCPs was important. This made patients feel valued by and gave value to the programme. Given these findings, nurturing the patient–HCP relationship is crucial for retention.

## Discussion

This systematic review, using meta-ethnographic synthesis, deepens understanding of the barriers and enablers that influence South Asian people’s participation in group health programmes. It is the first to move beyond describing barriers and facilitators to group education and exercise interventions for South Asian people living with LTCs, to developing new interpretive understandings that illuminate how and why these factors shape engagement.

Despite our initial focus on three LTCs (COPD, CHD and T2DM), we did not identify articles meeting our inclusion criteria for COPD, highlighting a paucity of qualitative data on the experiences of South Asian people with COPD in PR settings. While other reviews do exist in the area (eg, a review by Alamer *et al*)^44^, this is the first dedicated systematic review and meta-ethnography specifically examining interventions for South Asian people living with LTCs, allowing for more targeted policy and cross-contextual learning.

A key strength of this systematic review with meta-ethnography was the broad scope, enabling transferable insights and comprehensive understanding of engagement barriers. But data constraints prevented highlighting distinctions between communities, as studies either described samples simply as ‘South Asian’ or failed to explore cultural nuances. Additionally, we found no studies meeting inclusion criteria that specifically explored PR, representing a critical gap in the literature.

Our findings both align with and extend beyond earlier work.^45^ While existing literature acknowledges that cultural frameworks influence illness beliefs among South Asian communities,^11^ prior research has primarily treated these beliefs as contextual factors affecting diagnosis perception (eg, an analysis by Galdas and Kang)^30^. Our review specifically highlights their impact on perceived treatment effectiveness, positioning these beliefs as central obstacles directly impacting intervention engagement, rather than peripheral considerations.

Further, some studies interpret aspects like faith as a facilitator: emphasising how bodily care acts as a religious duty and therefore motivates health-seeking behaviours.^46^ Others, however, identify faith as a barrier, with religious fatalism reducing perceived agency and delaying help-seeking.^11 47^ This present review extends our understanding by demonstrating that faith can function as both a barrier and facilitator. Thus, HCPs may consider conducting culturally sensitive assessments to understand how patients’ religious beliefs may or may not be influencing their health decisions. Where appropriate, HCPs may also consider partnering with faith leaders and community organisations to deliver localised health messages in ways that align with religious teachings to increase intervention acceptability and uptake—rather than working against them. Other clinical implications may include: gender-segregated sessions and scheduling around religious observances.

Furthermore, we identified women-specific barriers (such as lacking autonomy and deprioritising their own health), resulting in unique disparities compared with Western counterparts.^48^,^50^ Understanding these gendered obstacles is crucial for designing interventions that are culturally sensitive and that empower women to take an active role in their health.

The implications of our findings are significant for clinicians and policymakers. Communication emerged as the most prominent barrier, with HCPs criticised for poor explanations and guidance, issues reflected in broader literature.^51 52^ As HCPs determine who receives care,^53^ culturally competent communication is essential to ensure equitable access and understanding. Improving communication has the potential to facilitate informed decision-making for patients and helps to build trust, particularly for patients from diverse cultural and religious backgrounds.

As evidenced, collaborative relationships with HCPs have the potential to be important facilitators. When HCPs engage with patients as active partners, it can increase confidence and engagement.^11 51^ This may be especially important for South Asian women who do not have the same life advantage as their Western counterparts.^49 54^

To facilitate more collaborative relationships between HCPs and patients, our findings suggest healthcare providers should: (1) implement longer appointments for patients with limited English proficiency; (2) increase workforce diversity to better reflect patient populations; (3) provide information in South Asian languages while recognising that spoken fluency does not always indicate reading ability; (4) develop consistent visual aids; and (5) deliver individualised treatment informed by cultural preferences, which has already been proven to increase engagement.^55^ Additionally, HCPs should recognise the powerful influence of community consensus on individual healthcare decisions, potentially leveraging respected community leaders to promote positive attitudes toward interventions.

Future research should address several critical gaps identified in this review. Studies exploring differences between distinct South Asian communities are needed to move beyond treating this population as homogeneous. Research should also investigate experiences of pulmonary rehabilitation specifically, as COPD and access to effective treatments remain problematic for South Asian communities.^5^ Finally, targeted research on South Asian women is warranted, as our findings suggest they face unique barriers. Addressing gaps and applying these recommendations can help healthcare systems provide fairer and more effective services for South Asian people living with long-term conditions.

### Limitations

This review is limited by the quality of the included studies and the translations. First, due to the paucity of research in this area, papers were not excluded based on quality, meaning that some lower-quality studies were included. While this could be viewed as a limitation, it was considered justified given the limited evidence base in this topic area. Second, the qualitative nature of the method introduces inherent researcher subjectivity that may affect the generalisability of conclusions. That said, the research teams commitment to systematic methods are expected to minimise this. Finally, the demographics of our LEAP members may have also limited this review (only representing the lived experience of COPD); this was due to the review being a smaller component of an NIHR-funded COPD project (NIHR205340).

A key strength of this review is its identification of unmet needs. By synthesising several data sets, we were able to summarise gaps that are prevalent across this population and generate new theory and insights about the barriers and facilitators of education and rehabilitation interventions for South Asian people with LTCs. Furthermore, input from our academic team, LEAP and the Research Advisory Group has enhanced the novelty and comprehensive nature of this review, offering a holistic perspective on the topic and providing evidence-based recommendations for future work. This contribution adds valuable insights to an otherwise limited body of qualitative research.

## Conclusions

This meta-ethnography synthesises how cultural and contextual factors shape engagement with group interventions among South Asian people with LTCs. Across studies, culture, faith, communication, perceptions of safety and relationships with HCPs emerge as key influences, functioning both as barriers and facilitators. Interventions that lack cultural sensitivity are less likely to be accessed or accepted, particularly by South Asian women, for whom cultural norms and competing responsibilities further constrain participation.

## Supplementary material

10.1136/bmjopen-2025-106694 online supplemental file 1

10.1136/bmjopen-2025-106694 online supplemental file 2

10.1136/bmjopen-2025-106694 online supplemental file 3

10.1136/bmjopen-2025-106694 online supplemental file 4

10.1136/bmjopen-2025-106694 online supplemental file 5

10.1136/bmjopen-2025-106694 online supplemental file 6

10.1136/bmjopen-2025-106694 online supplemental file 7

## Data Availability

All data relevant to the study are included in the article or uploaded as supplementary information.
