# Supplementary material for: Understanding barriers and facilitators to education and rehabilitation interventions for South Asian people with long-term conditions: a systematic review and meta-ethnography
Source: BMJ Open. 2026 Jan 13;16(1):e106694. doi: 10.1136/bmjopen-2025-106694 (PMC12815045; doi:10.1136/bmjopen-2025-106694)
Supplement: online supplemental file 2 [file bmjopen-16-1-s002.docx]

**Strategy 1: MEDLINE**

Step 1: COPD, Cardiovascular Disease, Diabetes

| COPD | exp Pulmonary Disease, Chronic Obstructive/ |
| --- | --- |
|  | Chronic Obstructive Pulmonary Disease.tw. |
|  | Chronic Obstructive Airway Disease.tw. |
|  | Chronic Obstructive Lung Disease.tw. |
|  | Pulmonary emphysema.tw. |
|  | Chronic bronchitis.tw. |
|  | (COPD OR COAD OR COBD OR AECOPD).tw. |
|  | Chronic Airflow Obstruction.tw. |
| CVD | exp Cardiovascular disease/ |
|  | exp Coronary artery disease/ |
|  | exp Coronary heart disease/ |
|  | exp Ischemic heart disease/ |
|  | exp Myocardial Ischemia/ |
|  | (((myocard* adj5 ischaemia OR myocard* adj5 ischemia).tw. |
|  | (isch?emi* adj5 heart).tw. |
|  | exp Atherosclerosis/ |
|  | Exp Coronary Artery Bypass/ |
|  | (myocard* adj5 infarct*).tw. |
|  | (heart adj5 infarct*).tw. |
|  | (heart adj5 attack*).tw. |
|  | angina.tw. |
|  | coronary adj5 (disease* OR bypass OR thrombo* OR angioplast*)).tw. |
|  | exp Angioplasty/ |
|  | angioplast*.tw. |
|  | ((coronary OR arterial) adj4 dilat*.tw. |
|  | exp Stents/ |
|  | exp Atherosclerosis/ |
|  | Acute coronary syndrome*.tw. |
|  | ACS.tw. |
| Diabetes | exp Diabetes Mellitus/ |
|  | Diabetes.tw. |
|  | Diabetes Mellitus.tw. |
|  | exp Diabetes Mellitus, Type 1/ |
|  | exp Diabetes Mellitus, Type 2/ |
|  | Type 2 diabetes mellitus.tw. |
|  | exp Diabetes, Gestational/ |
|  | exp Diabetes Complications/ |

Step 2: Group Interventions/Treatment

| Group Intervention/Treatment | exp Self-Management/ |
| --- | --- |
|  | (self-manag* OR self manag*).tw. |
|  | (self-manag* group OR self manag* group OR self-manag* program* OR self manage* program* OR self-manag* intervention OR self manag* intervention).tw. |
|  | exp Self Care OR Self-Care/ |
|  | exp Education/ |
|  | exp Patient Education as Topic/ |
|  | patient education.tw. |
|  | educat*.tw. |
|  | (patient education OR patient education group).tw. |
|  | exp Health Education/ |
|  | (health education).tw. |
|  | exp Health Promotion/ |
|  | (health adj3 education OR health adj3 promotion).tw. |
|  | self-car*.tw. |
|  | (management adj2 plan? OR management adj2 program*).tw. |
|  | exp Disease Management/ |
|  | disease management.tw. |
|  | (chronic disease management OR chronic illness management).tw. |
|  | (disease* adj2 management*).tw. |
|  | exp Managed Care Programs/ |
|  | pulmonary rehab*.tw. |
|  | Cardiac Rehabilitation/ |
|  | cardiac rehab*.tw. |
|  | diabetes education.tw. |
|  | behav* intervention.tw. |

Step 3: South Asian Populations

| South Asian Populations | Asia, Southern/ |
| --- | --- |
|  | South Asia*.tw. |
|  | Exp India/ |
|  | India*.tw. |
|  | Exp Sri Lanka/ |
|  | Sri Lanka*.tw. |
|  | Exp Pakistan/ |
|  | Pakistan*.tw. |
|  | Exp Bangladesh/ |
|  | Bangladesh*.tw. |
|  | Exp Nepal/ |
|  | Nepal*.tw. |
|  | exp Bhutan/ |
|  | Bhutan*.tw. |
|  | Exp Afghanistan/ |
|  | Afghan*.tw. |
|  | exp Maldives/ |
|  | Maldiv*.tw. |
|  | (ethnic minorit* OR racial minority*).tw. |
|  | Ethnic group.tw. |
|  | Asian People/ |
|  | British Asian*.tw. |
|  | Exp Asian/ |
|  | Asian American*.tw. |

Step 4: Qualitative Research

| Qualitaive Research | exp Qualitative Research/ |
| --- | --- |
|  | Nurs* Methodology Research/ |
|  | exp interview?/ |
|  | exp Attitude/ |
|  | exp Focus Groups/ |
|  | discourse analysis.tw. |
|  | content analysis.tw. |
|  | ethnographic research.tw. |
|  | ethnological research.tw. |
|  | ethnonursing research.tw. |
|  | constant comparative method.tw. |
|  | qualitative validity.tw. |
|  | purposive sample.tw. |
|  | observational method*.tw. |
|  | field stud*.tw. |
|  | theoretical sampl*.tw. |
|  | phenomenology.tw. |
|  | phenomenological research.tw. |
|  | life experience?.tw. |
|  | lived experience?.tw. |
|  | Narrativ*.tw. |
|  | Opinion*.tw. |
|  | Phenomengraph*.tw. |
|  | Hermeneutic*.tw. |
|  | Ethnograph*.tw. |
|  | Ethnomethodolog*.tw. |
|  | Exploratory stud*.tw. |
|  | Descriptive stud*.tw. |
|  | Autoethnograph*.tw. |
|  | (meta-syntheses?s OR metasynthes?s).tw |

**Strategy 2: PsychINFO**

Step 1: COPD, cardiovascular disease, diabetes

| COPD | Pulmonary Disease, Chronic Obstructive/ |
| --- | --- |
|  | exp lung disorders/ |
|  | Chronic Obstructive Pulmonary Disease.tw. |
|  | Chronic Obstructive Airway Disease.tw. |
|  | Chronic Obstructive Lung Disease.tw. |
|  | Pulmonary emphysema.tw. |
|  | Chronic bronchitis.tw. |
|  | (COPD or COAD or COBD or AECOPD).tw. |
|  | Chronic Airflow Obstruction.tw. |
| CVD | exp cardiovascular disorders/ |
|  | exp coronary heart disease/ |
|  | exp ischemia/ |
|  | exp myocardial infarctions/ |
|  | (myocard* adj5 ischaemia).mp. [mp=title, abstract, heading word, table of contents, key concepts, original title, tests & measures, mesh word] |
|  | (myocard* adj5 ischemia).tw. |
|  | (isch?emi* adj5 heart).tw. |
|  | exp atherosclerosis/ |
|  | exp cardiovascular disorders/ |
|  | (myocard* adj5 infarct*).tw. |
|  | (heart adj5 infarct*).tw. |
|  | (heart adj5 attack*).tw. |
|  | angina.tw. |
|  | (coronary adj5 (disease* or bypass or thrombo* or angioplast*)).tw. |
|  | exp angiography/ |
|  | angioplast*.tw. |
|  | ((coronary or arterial) adj4 dilat*).tw. |
|  | stents/ |
|  | Acute coronary syndrome*.tw. |
|  | ACS.tw. |
| Diabetes | exp diabetes mellitus/ |
|  | Diabetes.tw. |
|  | Diabetes Mellitus.tw. |
|  | exp diabetes mellitus/ |
|  | Type 2 diabetes mellitus.tw. |
|  | exp diabetes insipidus/ |
|  | exp diabetes insipidus/ |

Step 2: Group Interventions/Treatment

| Group Intervention/Treatment | exp self-management/ |
| --- | --- |
|  | (self-manag* or self manag*).tw. |
|  | (self-manag* group or self manag* group or self-manag* program* or self manage* program* or self-manag* intervention or self manag* intervention).tw. |
|  | Self Care.mp. or Self-Care/ [mp=title, abstract, heading word, table of contents, key concepts, original title, tests & measures, mesh word] |
|  | exp self-care skills/ |
|  | exp education students/ |
|  | exp client education/ |
|  | patient education.tw. |
|  | educat*.tw. |
|  | (patient education or patient education group).tw. |
|  | exp health education/ |
|  | health education.tw. |
|  | exp health promotion/ |
|  | (((health adj3 education) or health) adj3 promotion).tw. |
|  | self-car*.tw. |
|  | (((management adj2 plan?) or management) adj2 program*).tw. |
|  | exp disease management/ |
|  | disease management.tw. |
|  | (chronic disease management or chronic illness management).tw. |
|  | (disease* adj2 management*).tw. |
|  | exp managed care/ |
|  | pulmonary rehab*.tw. |
|  | Cardiac Rehabilitation/ |
|  | cardiac rehab*.tw. |
|  | diabetes education.tw. |
|  | behav* intervention.tw. |

Step 3: South Asian Populations

| South Asian Populations | Asia, Southern/ |
| --- | --- |
|  | South Asia*.tw. |
|  | exp american indians/ |
|  | India*.tw. |
|  | Sri Lanka.tw. |
|  | Pakistan*.tw. |
|  | Bangladesh*.tw. |
|  | Nepal*.tw. |
|  | Bhutan*.tw. |
|  | Afghan*.tw. |
|  | Maldiv*.tw. |
|  | (ethnic minorit* or racial minority*).tw. |
|  | Ethnic group.tw. |
|  | Asian People/ |
|  | British Asian*.tw. |
|  | exp asians/ |
|  | Asian American*.tw. |

Step 4: Qualitative Research

| Qualitative Research | exp Qualitative Research/ |
| --- | --- |
|  | Nurs* Methodology Research/ |
|  | exp interview schedules/ |
|  | exp attitude similarity/ |
|  | Focus Groups/ |
|  | discourse analysis.tw. |
|  | content analysis.tw. |
|  | ethnographic research.tw. |
|  | ethnological research.tw. |
|  | ethnonursing research.tw. |
|  | constant comparative method.tw. |
|  | qualitative validity.tw. |
|  | purposive sample.tw. |
|  | observational method*.tw. |
|  | field stud*.tw. |
|  | theoretical sampl*.tw. |
|  | phenomenology.tw. |
|  | phenomenological research.tw. |
|  | life experience?.tw. |
|  | lived experience?.tw. |
|  | Narrativ*.tw. |
|  | Opinion*.tw. |
|  | Phenomengraph*.tw. |
|  | Hermeneutic*.tw. |
|  | Ethnograph*.tw. |
|  | Ethnomethodolog*.tw. |
|  | Exploratory stud*.tw. |
|  | Descriptive stud*.tw. |
|  | Autoethnograph*.tw. |
|  | (meta-syntheses?s or metasynthes?s).tw. |

**Strategy 3: CINAHL**

Step 1: COPD, cardiovascular disease, diabetes

| COPD | (MH "Pulmonary Disease, Chronic Obstructive+") |
| --- | --- |
|  | Chronic Obstructive Pulmonary Disease.tw. |
|  | Chronic Obstructive Airway Disease.tw. |
|  | Chronic Obstructive Lung Disease.tw. |
|  | Pulmonary emphysema.tw. |
|  | Chronic bronchitis.tw. |
|  | (COPD OR COAD OR COBD OR AECOPD).tw. |
|  | Chronic Airflow Obstruction.tw. |
| CVD | (MH "Cardiovascular Diseases+") |
|  | (MH "Coronary Arteriosclerosis") |
|  | (MH "Coronary Disease+") |
|  | (MH "Myocardial Ischemia+") |
|  | (((myocard* adj5 ischaemia OR myocard* adj5 ischemia).tw. |
|  | (isch?emi* adj5 heart).tw. |
|  | (MH "Atherosclerosis") OR (MH "Coronary Arteriosclerosis") OR (MH "Intracranial Arteriosclerosis+") |
|  | (MH "Coronary Artery Bypass+") |
|  | (myocard* adj5 infarct*).tw. |
|  | (heart adj5 infarct*).tw. |
|  | (heart adj5 attack*).tw. |
|  | angina.tw. |
|  | coronary adj5 (disease* OR bypass OR thrombo* OR angioplast*)).tw. |
|  | (MH "Angioplasty+") |
|  | angioplast*.tw. |
|  | ((coronary OR arterial) adj4 dilat*.tw. |
|  | (MH "Stents+") |
|  | (MH "Atherosclerosis") OR (MH "Intracranial Arteriosclerosis+") OR (MH "Coronary Arteriosclerosis") |
|  | Acute coronary syndrome*.tw. |
|  | ACS.tw. |
| Diabetes | (MH "Diabetes Mellitus+") |
|  | Diabetes.tw. |
|  | diabetes |
|  | Diabetes Mellitus.tw. |
|  | Diabetes Mellitus |
|  | (MH "Diabetes Mellitus, Type 1+") OR (MH "Diabetes Mellitus, Type 2") |
|  | Type 2 diabetes mellitus.tw. |
|  | (MH "Diabetes Mellitus, Gestational") |
|  | "Diabetes Complications" |

Step 2: Group Interventions/Treatment

| Group Intervention/Treatment | (MH "Self Care+") OR "Self-Management" |
| --- | --- |
|  | (self-manag* OR self manag*).tw. |
|  | (self-manag* group OR self manag* group OR self-manag* program* OR self manage* program* OR self-manag* intervention OR self manag* intervention).tw. |
|  | (MH "Self Care+") OR "Self Care OR Self-Care" |
|  | (MH "Education+") |
|  | (MH "Patient Education+") OR "Patient Education as Topic" |
|  | patient education.tw. |
|  | educat*.tw. |
|  | (patient education OR patient education group).tw. |
|  | (MH "Health Education+") |
|  | (health education).tw. |
|  | (MH "Health Promotion") |
|  | (health adj3 education OR health adj3 promotion).tw. |
|  | self-car*.tw. |
|  | (management adj2 plan? OR management adj2 program*).tw. |
|  | (MH "Disease Management+") |
|  | disease management.tw. |
|  | (chronic disease management OR chronic illness management).tw. |
|  | (disease* adj2 management*).tw. |
|  | (MH "Managed Care Programs+") |
|  | pulmonary rehab*.tw. |
|  | Cardiac Rehabilitation/ |
|  | cardiac rehab*.tw. |
|  | diabetes education.tw. |
|  | behav* intervention.tw. |

Step 3: South Asian Populations

| South Asian Populations | Asia, Southern/ |
| --- | --- |
|  | South Asia*.tw. |
|  | (MH "India") |
|  | India*.tw. |
|  | (MH "Sri Lanka") OR (MH "Sri Lankans") |
|  | Sri Lanka*.tw. |
|  | (MH "Pakistan") OR (MH "Pakistanis") |
|  | Pakistan*.tw. |
|  | (MH "Bangladesh") OR (MH "Bangladeshi Persons") |
|  | Bangladesh*.tw. |
|  | (MH "Nepali Persons") OR (MH "Himalayas") |
|  | Nepal*.tw. |
|  | (MH "Bhutan") OR (MH "Himalayas") |
|  | Bhutan*.tw. |
|  | (MH "Afghanistan") OR (MH "Afghan Persons") OR (MH "Asia, Central+") |
|  | Afghan*.tw. |
|  | (MH "Maldives") |
|  | Maldiv*.tw. |
|  | (ethnic minorit* OR racial minority*).tw. |
|  | Ethnic group.tw. |
|  | Asian People/ |
|  | British Asian*.tw. |
|  | (MH "Asian Canadians+") OR (MH "Asian Americans+") OR (MH "Asian Indians+") OR (MH "Asian Indian Americans") OR (MH "South Asian Americans+") OR (MH "East Asian Americans+") OR (MH "Southeast Asian Americans+") OR (MH "South Asians+") OR (MH "East Asians+") OR (MH "Southeast Asians+") OR (MH "Central Asians+") OR (MH "Asians+") OR (MH "Malays") |
|  | Asian American*.tw. |

Step 4: Qualitative Research

| Qualitaive Research | (MH "Qualitative Studies+") OR "Qualitative Research/" |
| --- | --- |
|  | Nurs* Methodology Research/ |
|  | (MH "Interviews+") |
|  | (MH "Attitude+") |
|  | (MH "Focus Groups") |
|  | discourse analysis |
|  | content analysis |
|  | ethnographic research |
|  | ethnological research |
|  | ethnonursing research |
|  | constant comparative method |
|  | qualitative validity |
|  | purposive sample |
|  | observational method* |
|  | field stud*.tw. |
|  | theoretical sampl* |
|  | phenomenology |
|  | phenomenological research |
|  | life experience? |
|  | lived experience? |
|  | Narrativ* |
|  | Opinion* |
|  | Phenomengraph* |
|  | Hermeneutic* |
|  | Ethnograph* |
|  | Ethnomethodolog* |
|  | Exploratory stud* |
|  | Descriptive stud* |
|  | Autoethnograph* |
|  | (meta-syntheses?s OR metasynthes?s) |

**Strategy 4: CENTRAL**

Step 1: COPD, cardiovascular disease, diabetes

| COPD | MeSH descriptor: [Pulmonary Disease, Chronic Obstructive] explode all trees |
| --- | --- |
|  | ("Chronic Obstructive Pulmonary Disease" OR "Chronic Obstructive Airway Disease" OR "Chronic Obstructive Lung Disease" OR "Pulmonary emphysema" OR "Chronic bronchitis"):ti,ab,kw |
|  | ("COPD" OR "COAD" OR "COBD" OR "AECOPD"):ti,ab,kw |
|  | (Chronic Airflow Obstruction):ti,ab,kw |
| CVD | MeSH descriptor: [Cardiovascular Diseases] explode all trees |
|  | MeSH descriptor: [Coronary Artery Disease] explode all trees |
|  | MeSH descriptor: [Coronary Disease] explode all trees |
|  | MeSH descriptor: [Myocardial Ischemia] explode all trees |
|  | MeSH descriptor: [Myocardial Ischemia] explode all trees |
|  | (myocard* NEAR/5 ischaemia OR myocard* NEAR/5 ischemia):ti,ab,kw |
|  | (isch?emi* near/5 heart):ti,ab,kw |
|  | MeSH descriptor: [Atherosclerosis] explode all trees |
|  | MeSH descriptor: [Coronary Artery Bypass] explode all trees |
|  | (myocard* NEXT/5 infarct*):ti,ab,kw |
|  | (heart NEXT/5 infarct*):ti,ab,kw |
|  | (heart NEXT/5 attack*):ti,ab,kw |
|  | (angina):ti,ab,kw |
|  | (coronary NEXT/5 disease* OR bypass OR thrombo* OR angioplast*):ti,ab,kw |
|  | MeSH descriptor: [Angioplasty] explode all trees |
|  | (angioplast*):ti,ab,kw |
|  | ("coronary" OR "arterial" NEXT/5 dilat*):ti,ab,kw |
|  | MeSH descriptor: [Stents] explode all trees |
|  | MeSH descriptor: [Atherosclerosis] explode all trees |
|  | MeSH descriptor: [Acute Coronary Syndrome] explode all trees |
|  | (ACS):ti,ab,kw |
|  | MeSH descriptor: [Diabetes Mellitus] explode all trees |
| Diabetes | ("Diabetes Mellitus" OR "Diabetes" OR "Type 2 Diabetes Mellitus"):ti,ab,kw |
|  | MeSH descriptor: [Diabetes Mellitus, Type 1] explode all trees |
|  | MeSH descriptor: [Diabetes Mellitus, Type 2] explode all trees |
|  | MeSH descriptor: [Diabetes, Gestational] explode all trees |
|  | MeSH descriptor: [Diabetes Complications] explode all trees |

Step 2: Group Interventions/Treatment

| Group Intervention/Treatment | MeSH descriptor: [Self-Management] explode all trees |
| --- | --- |
|  | (self-manag* OR self manag* OR self-manag* group OR self manag* group OR self-manag* program* OR self manage* program* OR self-manag* intervention OR self manag* intervention):ti,ab,kw |
|  | MeSH descriptor: [Self Care] explode all trees |
|  | MeSH descriptor: [Education] explode all trees |
|  | MeSH descriptor: [Patient Education as Topic] explode all trees |
|  | (patient education):ti,ab,kw |
|  | (educat*):ti,ab,kw |
|  | (patient education OR patient education group):ti,ab,kw |
|  | MeSH descriptor: [Health Educators] in all MeSH products |
|  | (health education):ti,ab,kw |
|  | MeSH descriptor: [Health Educators] explode all trees |
|  | (health NEXT/3 education OR health NEXT/3 promotion):ti,ab,kw |
|  | (Self-car*):ti,ab,kw |
|  | (management next/2 plan? OR management next/2 program*):ti,ab,kw |
|  | MeSH descriptor: [Disease Management] explode all trees |
|  | (disease management):ti,ab,kw |
|  | (chronic disease management OR chronic illness management):ti,ab,kw |
|  | (disease* NEXT/2 management*):ti,ab,kw |
|  | MeSH descriptor: [Managed Care Programs] explode all trees |
|  | (pulmonary rehab*):ti,ab,kw |
|  | (Cardiac Rehabilitation):ti,ab,kw |
|  | (Cardiac Rehab*):ti,ab,kw |
|  | (diabetes education):ti,ab,kw |
|  | (behav* intervention):ti,ab,kw |

Step 3: South Asian Populations

| South Asian Populations | (Asia Southern):ti,ab,kw |
| --- | --- |
|  | (South Asia*):ti,ab,kw |
|  | MeSH descriptor: [India] explode all trees |
|  | (India*):ti,ab,kw |
|  | MeSH descriptor: [Sri Lanka] explode all trees |
|  | (Sri Lanka*):ti,ab,kw |
|  | MeSH descriptor: [Pakistan] explode all trees |
|  | (pakistan):ti,ab,kw |
|  | MeSH descriptor: [Bangladesh] explode all trees |
|  | MeSH descriptor: [Nepal] explode all trees |
|  | (nepal):ti,ab,kw |
|  | MeSH descriptor: [Bhutan] explode all trees |
|  | (Bhutan):ti,ab,kw |
|  | MeSH descriptor: [Afghanistan] explode all trees |
|  | (Afghan*):ti,ab,kw |
|  | MeSH descriptor: [Maldives] explode all trees |
|  | (maldiv*):ti,ab,kw |
|  | (ethnic minorit* OR racial minority*):ti,ab,kw |
|  | (ethnic group):ti,ab,kw |
|  | (Asian people):ti,ab,kw |
|  | (british asian):ti,ab,kw |
|  | MeSH descriptor: [Asian] explode all trees |
|  | (Asian American*):ti,ab,kw |

Step 4: Qualitative Research

| Qualitaive Research | MeSH descriptor: [Qualitative Research] explode all trees |
| --- | --- |
|  | (Nurs* Methodology Research):ti,ab,kw |
|  | MeSH descriptor: [Interview] in all MeSH products |
|  | (interview?):ti,ab,kw |
|  | MeSH descriptor: [Attitude] explode all trees |
|  | MeSH descriptor: [Focus Groups] explode all trees |
|  | (discourse analysis):ti,ab,kw |
|  | (content analysis):ti,ab,kw |
|  | (ethno* research):ti,ab,kw |
|  | (constant comparative method):ti,ab,kw |
|  | (qualitative validity):ti,ab,kw |
|  | (purposive sample):ti,ab,kw |
|  | (observational method*):ti,ab,kw |
|  | (field stud*):ti,ab,kw |
|  | (theoretical sampl*):ti,ab,kw |
|  | (phenomenology):ti,ab,kw |
|  | (phenomenological research):ti,ab,kw |
|  | (life experience?):ti,ab,kw |
|  | (lived experience?):ti,ab,kw |
|  | (Narrativ*):ti,ab,kw |
|  | (Opinion*):ti,ab,kw |
|  | (Phenomengraph*):ti,ab,kw |
|  | (Hermeneutic*):ti,ab,kw |
|  | (Ethnograph*):ti,ab,kw |
|  | (Ethnomethodolog*):ti,ab,kw |
|  | (Exploratory stud*):ti,ab,kw |
|  | (Descriptive stud*):ti,ab,kw |
|  | (Autoethnograph*):ti,ab,kw |
|  | (meta-syntheses?s OR metasynthes?s):ti,ab,kw |

**Strategy 5: EMBASE**

Step 1: COPD, cardiovascular disease, diabetes

| COPD | Pulmonary Disease, Chronic Obstructive/ |
| --- | --- |
|  | exp lung disorders/ |
|  | Chronic Obstructive Pulmonary Disease.tw. |
|  | Chronic Obstructive Airway Disease.tw. |
|  | Chronic Obstructive Lung Disease.tw. |
|  | Pulmonary emphysema.tw. |
|  | Chronic bronchitis.tw. |
|  | (COPD or COAD or COBD or AECOPD).tw. |
|  | Chronic Airflow Obstruction.tw. |
| CVD | exp cardiovascular disorders/ |
|  | exp coronary heart disease/ |
|  | exp ischemia/ |
|  | exp myocardial infarctions/ |
|  | (myocard* adj5 ischaemia).mp. [mp=title, abstract, heading word, table of contents, key concepts, original title, tests & measures, mesh word] |
|  | (myocard* adj5 ischemia).tw. |
|  | (isch?emi* adj5 heart).tw. |
|  | exp atherosclerosis/ |
|  | exp cardiovascular disorders/ |
|  | (myocard* adj5 infarct*).tw. |
|  | (heart adj5 infarct*).tw. |
|  | (heart adj5 attack*).tw. |
|  | angina.tw. |
|  | (coronary adj5 (disease* or bypass or thrombo* or angioplast*)).tw. |
|  | exp angiography/ |
|  | angioplast*.tw. |
|  | ((coronary or arterial) adj4 dilat*).tw. |
|  | stents/ |
|  | Acute coronary syndrome*.tw. |
|  | ACS.tw. |
| Diabetes | exp diabetes mellitus/ |
|  | Diabetes.tw. |
|  | Diabetes Mellitus.tw. |
|  | exp diabetes mellitus/ |
|  | Type 2 diabetes mellitus.tw. |
|  | exp diabetes insipidus/ |
|  | exp diabetes insipidus/ |

Step 2: Group Interventions/Treatment

| Group Intervention/Treatment | exp self-management/ |
| --- | --- |
|  | (self-manag* or self manag*).tw. |
|  | (self-manag* group or self manag* group or self-manag* program* or self manage* program* or self-manag* intervention or self manag* intervention).tw. |
|  | Self Care.mp. or Self-Care/ [mp=title, abstract, heading word, table of contents, key concepts, original title, tests & measures, mesh word] |
|  | exp self-care skills/ |
|  | exp education students/ |
|  | exp client education/ |
|  | patient education.tw. |
|  | educat*.tw. |
|  | (patient education or patient education group).tw. |
|  | exp health education/ |
|  | health education.tw. |
|  | exp health promotion/ |
|  | (((health adj3 education) or health) adj3 promotion).tw. |
|  | self-car*.tw. |
|  | (((management adj2 plan?) or management) adj2 program*).tw. |
|  | exp disease management/ |
|  | disease management.tw. |
|  | (chronic disease management or chronic illness management).tw. |
|  | (disease* adj2 management*).tw. |
|  | exp managed care/ |
|  | pulmonary rehab*.tw. |
|  | Cardiac Rehabilitation/ |
|  | cardiac rehab*.tw. |
|  | diabetes education.tw. |
|  | behav* intervention.tw. |

Step 3: South Asian Populations

| South Asian Populations | Asia, Southern/ |
| --- | --- |
|  | South Asia*.tw. |
|  | exp american indians/ |
|  | India*.tw. |
|  | Sri Lanka.tw. |
|  | Pakistan*.tw. |
|  | Bangladesh*.tw. |
|  | Nepal*.tw. |
|  | Bhutan*.tw. |
|  | Afghan*.tw. |
|  | Maldiv*.tw. |
|  | (ethnic minorit* or racial minority*).tw. |
|  | Ethnic group.tw. |
|  | Asian People/ |
|  | British Asian*.tw. |
|  | exp asians/ |
|  | Asian American*.tw. |

Step 4: Qualitative Research

| Qualitaive Research | exp Qualitative Research/ |
| --- | --- |
|  | Nurs* Methodology Research/ |
|  | exp interview schedules/ |
|  | exp attitude similarity/ |
|  | Focus Groups/ |
|  | discourse analysis.tw. |
|  | content analysis.tw. |
|  | ethnographic research.tw. |
|  | ethnological research.tw. |
|  | ethnonursing research.tw. |
|  | constant comparative method.tw. |
|  | qualitative validity.tw. |
|  | purposive sample.tw. |
|  | observational method*.tw. |
|  | field stud*.tw. |
|  | theoretical sampl*.tw. |
|  | phenomenology.tw. |
|  | phenomenological research.tw. |
|  | life experience?.tw. |
|  | lived experience?.tw. |
|  | Narrativ*.tw. |
|  | Opinion*.tw. |
|  | Phenomengraph*.tw. |
|  | Hermeneutic*.tw. |
|  | Ethnograph*.tw. |
|  | Ethnomethodolog*.tw. |
|  | Exploratory stud*.tw. |
|  | Descriptive stud*.tw. |
|  | Autoethnograph*.tw. |
|  | (meta-syntheses?s or metasynthes?s).tw. |

**Strategy 6: Applied Social Sciences Index and Abstracts (SEARCH TERMS REDUCED DUE TO OVERWHELMING DATA SIZE)**

Step 1: COPD, cardiovascular disease, diabetes

| COPD | Chronic Obstructive Pulmonary Disease |
| --- | --- |
|  | COPD |
| CVD | cardiovascular disease |
|  | CVD |
| Diabetes | Diabetes OR Diabetes Mellitus |

Step 2: Group Interventions/Treatment

| Group Intervention/Treatment | self-manag* OR self manag* |
| --- | --- |
|  | [educat*](https://www.proquest.com/recentsearches.recentsearchtabview.recentsearchesgridview.scrolledrecentsearchlist.checkdbssearchlink:rerunsearch/77388932DCD1446DPQ/None/$N?t:ac=RecentSearches) |
|  | pulmonary rehab* |

Step 3: South Asian Populations

| Population | [South* Asia*](https://www.proquest.com/recentsearches.recentsearchtabview.recentsearchesgridview.scrolledrecentsearchlist.checkdbssearchlink:rerunsearch/C30A1C941C054169PQ/None/$N?t:ac=RecentSearches) |
| --- | --- |

Step 4: Qualitative Research

| Research | Qualitative Research |
| --- | --- |
