# Supplementary material for: Understanding barriers and facilitators to education and rehabilitation interventions for South Asian people with long-term conditions: a systematic review and meta-ethnography
Source: BMJ Open. 2026 Jan 13;16(1):e106694. doi: 10.1136/bmjopen-2025-106694 (PMC12815045; doi:10.1136/bmjopen-2025-106694)
Supplement: online supplemental file 3 [file bmjopen-16-1-s003.docx]

**Table 1:** JBI Summary

| **ARTICLE** | **D1** | **D2** | **D3** | **D4** | **D5** | **D6** | **D7** | **D8** | **D9** | **D10** | **SCORE** |
| --- | --- | --- | --- | --- | --- | --- | --- | --- | --- | --- | --- |
| [1] | Y | Y | Y | Y | Y | Y | Y | Y | Y | Y | 10 |
| [2] | Y | Y | Y | Y | Y | N | N | Y | Y | Y | 8 |
| [3] | Y | Y | Y | Y | Y | N | N | Y | Y | Y | 8 |
| [4] | Y | Y | Y | Y | Y | N | N | Y | Y | Y | 8 |
| [5] | Y | Y | Y | Y | Y | N | N | Y | Y | Y | 8 |
| [6] | - | Y | Y | Y | Y | N | Y | Y | Y | Y | 8 |
| [7] | - | Y | Y | Y | Y | - | Y | Y | Y | Y | 8 |
| [8] | Y | Y | Y | Y | Y | Y | N | Y | Y | Y | 8 |
| [9] | Y | Y | Y | - | - | N | Y | Y | Y | Y | 7 |
| [10] | - | Y | Y | Y | Y | N | N | Y | Y | Y | 7 |
| [11] | - | Y | Y | Y | Y | N | N | Y | Y | Y | 7 |
| [12] | N | Y | Y | Y | Y | N | N | Y | Y | Y | 7 |
| [13] | - | Y | Y | Y | Y | N | N | Y | Y | Y | 7 |
| [14] | N | Y | Y | Y | Y | N | N | Y | Y | Y | 7 |
| [15] | N | - | Y | Y | Y | N | N | Y | Y | Y | 6 |
| [16] | Y | Y | Y | - | - | N | N | Y | - | Y | 5 |
| [17] | N | Y | - | - | - | N | N | Y | Y | Y | 4 |

References

[1]. Dilla, D., Ian, J., Martin, J., Michelle, H., & Felicity, A. (2020). “I don’t do it for myself, I do it for them”: A grounded theory study of South Asians’ experiences of making lifestyle change after myocardial infarction. Journal of Clinical Nursing, 29(19-20), 3687-3700.

[2]. Darr, A., Astin, F., & Atkin, K. (2008). Causal attributions, lifestyle change, and coronary heart disease: illness beliefs of patients of South Asian and European origin living in the United Kingdom. Heart & Lung, 37(2), 91-104.

[3]. Galdas, P. M., & Kang, H. B. K. (2010). Punjabi Sikh patients’ cardiac rehabilitation experiences following myocardial infarction: a qualitative analysis. Journal of clinical nursing, 19(21‐22), 3134-3142.

[4]. Galdas, P. M., Oliffe, J. L., Kang, H. B. K., & Kelly, M. T. (2012). Punjabi Sikh Patients’ Perceived Barriers to Engaging in Physical Exercise Following Myocardial Infarction. Public Health Nursing, 29(6), 534-541.

[5]. Galdas, P. M., Oliffe, J. L., Wong, S. T., Ratner, P. A., Johnson, J. L., & Kelly, M. T. (2012). Canadian Punjabi Sikh men’s experiences of lifestyle changes following myocardial infarction: cultural connections. Ethnicity & health, 17(3), 253-266.

[6]. Webster, R. A., Thompson, D. R., & Mayou, R. A. (2002). The experiences and needs of Gujarati Hindu patients and partners in the first month after a myocardial infarction. European Journal of Cardiovascular Nursing, 1(1), 69-76.

[7]. Bandyopadhyay, M. (2021). Gestational diabetes mellitus: a qualitative study of lived experiences of South Asian immigrant women and perspectives of their health care providers in Melbourne, Australia. BMC Pregnancy and Childbirth, 21, 1-12.

[8]. Astin, F., Atkin, K., & Darr, A. (2008). Family support and cardiac rehabilitation: a comparative study of the experiences of South Asian and White-European patients and their carer’s living in the United Kingdom. European Journal of Cardiovascular Nursing, 7(1), 43-51.

[9]. Grewal, K., Leung, Y. W., Safai, P., Stewart, D. E., Anand, S., Gupta, M., ... & Grace, S. L. (2010). Access to cardiac rehabilitation among South-Asian patients by referral method: a qualitative study. Rehabilitation Nursing Journal, 35(3), 106-112.

[10]. Patel, N., Stone, M. A., Hadjiconstantinou, M., Hiles, S., Troughton, J., Martin-Stacey, L., ... & Khunti, K. (2015). Using an interactive DVD about type 2 diabetes and insulin therapy in a UK South Asian community and in patient education and healthcare provider training. Patient education and counselling, 98(9), 1123-1130.

[11]. Banerjee, A. T., Grace, S. L., Thomas, S. G., & Faulkner, G. (2010). Cultural factors facilitating cardiac rehabilitation participation among Canadian South Asians: a qualitative study. Heart & Lung, 39(6), 494-503.

[12]. Chauhan, U., Baker, D., Lester, H., & Edwards, R. (2010). Exploring uptake of cardiac rehabilitation in a minority ethnic population in England: a qualitative study. European Journal of Cardiovascular Nursing, 9(1), 68-74.

[13]. Coe, C., & Boardman, S. (2008). From temple to table: an innovative community health and lifestyle intervention aimed at a South Asian community. Ethnicity and Inequalities in Health and Social Care, 1(2), 44-51.

[14]. Visram, S., Crosland, A., Unsworth, J., & Long, S. (2008). Engaging women from South Asian communities in cardiac rehabilitation. International Journal of Therapy and Rehabilitation, 15(7), 298-305.

[15]. Jolly, K., Taylor, R., Lip, G. Y., Greenfield, S., Raftery, J., Mant, J., ... & Stevens, A. (2007). The Birmingham Rehabilitation Uptake Maximisation Study (BRUM). Home-based compared with hospital-based cardiac rehabilitation in a multi-ethnic population: cost-effectiveness and patient adherence. Health Technology Assessment (Winchester, England), 11(35), 1-118.

[16]. Jolly, K., Greenfield, S. M., & Hare, R. (2004). Attendance of ethnic minority patients in cardiac rehabilitation. Journal of Cardiopulmonary Rehabilitation and Prevention, 24(5), 308-312.

[17]. Jones, M., Jolly, K., Raftery, J., Lip, G. Y., & Greenfield, S. (2007). ‘DNA ‘may not mean ‘did not participate’: a qualitative study of reasons for non-adherence at home-and centre-based cardiac rehabilitation. Family practice, 24(4), 343-357.
