# Supplementary material for: Understanding barriers and facilitators to education and rehabilitation interventions for South Asian people with long-term conditions: a systematic review and meta-ethnography
Source: BMJ Open. 2026 Jan 13;16(1):e106694. doi: 10.1136/bmjopen-2025-106694 (PMC12815045; doi:10.1136/bmjopen-2025-106694)
Supplement: online supplemental file 4 [file bmjopen-16-1-s004.docx]

**No. Article**

1 Dilla et al., 2020

2 Darr et al., 2008

3 Galdas & Kang, 2010

4 Galdas et al., 2012a

5 Galdas et al., 2012b

6 Webster et al., 2002

7 Bandyopadhyay, 2021

8 Astin et al., 2008

9 Grewal et al., 2010

10 Patel et al., 2015

11 Banerjee et al., 2010

12 Chauhan et al., 2010

13 Coe & Boardman, 2008

14 Visram et al., 2008

15 Jolly et al., 2007

16 Jolly et al., 2004

17 Jones et al., 2007

**Reciprocal Analysis**

*Language and Communication: Reciprocal Synthesis of papers 3, 5, 6, 8, 9, 12, 13, 14, 15, 16*

*Paper 3 showed how many south Asian people -- despite knowing English -- tend to be unable to gain an adequate depth of knowledge about [group] treatments as their limited language skills restrict their access to helpful information. While some hospitals had better access to Punjabi-speaking doctors, resource issues meant not all patients could access this service, suggesting this method to not currently be feasible without further funding and supply. Paper 5 also evidenced poor medical and health knowledge within the south Asian community (lack of experience with the exercise apparatus e.g., bikes, combined with language barriers prohibiting learning) generating apprehension around participating in group exercise which later led to drop-out and incompletion of the programme. Paper 6 builds on these issues highlighting a sense of frustration, with language barriers, and by association, knowledge barriers, often being seen as an insurmountable challenge. Paper 8 showed a popular solution to poor communication to be English-speaking children, who played crucial roles in facilitating information exchange. Findings from paper 9 suggest this may be a better medium than educational pamphlets that were deemed ineffective causing many to be unaware of the necessity of rehabilitation programs. Ultimately there was a strong preference for in-person face-to-face discussions over written or non-verbal mediums. That said, some participants appreciated the universal referral method, feeling it was well-organised. However regardless of communication methods, the decision to enrol in rehabilitation groups ultimately tended to be a personal decision. Paper 11 also states that important information tended to be poorly explained by healthcare actors, and through mediums that tended to be difficult for south Asian audiences to understand. This was exacerbated by HCP's having little dedicated time to effectively explain diagnosis and recovery. Additionally, leaflets provided by healthcare services were not considered sufficient nor an adequate means of communication for people experiencing such illness, which for most tended to be their first time. Paper 12 continues to explore the theme of inadequate knowledge and communication, presenting intriguing findings on the use of interpreters. Despite some hospitals offering interpreting services, generally patients still struggled to describe their symptoms accurately using this service. Generally healthcare professionals (HCP’s) tended to not engage directly with non-English speaking patients, relying instead on interpreters or English-speaking family members. Assumptions that some patients could not speak English were harmful, leading to a lack of direct engagement. Atypical presentations of symptoms and breathlessness further complicated communication, often making important discussions inaccessible without further support in some capacity. Paper 13 builds on this emphasising that systemic issues, particularly those related to language, hinder South Asian patients from visiting their GP’s. These barriers can deter south Asian people from seeking essential medical care, accentuating the criticality of more inclusive and accessible healthcare services. That said, healthcare services must approach methods carefully as Paper 14 found that interpreters can often be distracting to both service users and HCP’s, suggesting familiar people to be more effective in facilitating communication with south Asian people. Finally, Paper 15 also highlighted the importance of improving communication methods, noting that the inflexibility of current systems often leads to misunderstandings, including frequent confusion around scheduling sessions, resulting in many missed appointments.*

*Family and Social Support: Reciprocal Synthesis of papers 1, 3, 4, 5, 8, 12, 13, 14, 15*

*Findings from paper 1 show that ‘in times of crisis’ family support is critical, particularly in motivating south Asian people to make healthy lifestyle choices. South Asian patients told researchers that their culture places emphasis on supporting family members, suggesting that it is a responsibility, rather than a choice. Because of this cultural obligation, family members are likely to make healthier choices to protect their family, rather than doing so to benefit themselves. Paper 3 also investigated motivators to group treatments, suggesting shared experience to be key in encouraging south Asian patients to participate in cardiac rehabilitation. Fostering relationships between ‘like-minded’ patients was felt to create a sense of belonging and recognition. This was deemed the ‘most important part of the programme’, indicating peer support and diverse, representative audiences to be an effective and inspiring method to raising engagement and retention within group programmes. Paper 4 builds on this collectivist behaviour discussing how changes must be actively supported by the entire family to be effectively implemented and maintained. This was particularly important for south Asian males as they tend to rely on female family members e.g., wives and daughters, for meal preparation and must have their support to enforce changes learnt in cardiac rehabilitation. Paper 5 builds on this sense of reliance, as south Asian migrants felt that a lack of ‘walking companions’ reduced their desire to exercise. Additionally, they felt that forming friendships outside of South Asia was difficult, partly as other cultures tended to have different social traditions to their usual daily walks with friends. Paper 8 however shows how excessive support, particularly from family, can be problematic. While support can be beneficial, some children concealed diagnoses or negative health news from their families to protect their mental health. This can lead to patients being unaware of their conditions, preventing necessary lifestyle changes and attendance at potentially life-changing appointments or programmes. Additionally, out of concern, children may restrict family members from overexerting themselves, leading to misplaced perceptions and apprehensions that group exercise classes are too strenuous and potentially harmful. This was identified as the primary reason for lower attendance among South Asian people. A notable finding is the gender disparity in health support. South Asian families are less likely to adjust for a woman’s health needs, whereas they readily change routines for an unwell man. Consequently, female South Asian patients receive less family support compared to males when facing health issues. Paper 12 reciprocates this potentially dangerous reliance on patients children as due to acting as someone’s only method of communication doctors may begin to speaking exclusively with the children, leaving patients to feel excluded or disengaged from their treatment or care plan. While paper 13 does not acknowledge the role of the child, like papers 3 and 5, it indicates peer support to be a major motivation for the participation and retention of south Asian people in group treatment settings. Future group treatments are advised to make peer support their central responsibility to make services more enjoyable and less intimidating for south Asian patients. Paper 14 also addresses female-centric issues, highlighting how many female patients are disallowed certain treatments due to ‘cultural reservations’. This is particularly concerning because those who accessed these treatments experienced improvements in health, knowledge, and social integration, generally feeling more capable of incorporating new practices into their lives. Paper 15 builds on the narrative of family, friendship, and community support, presenting its significant influence over south Asian people with LTC’s, particularly one’s that require dietary changes. Collective community efforts could effectively increase engagement in health-promoting activities, such as cardiac rehabilitation, among South Asian people, particularly women, who have been identified as a care gap.*

*Cultural and Religious Factors: Reciprocal Synthesis of papers 1, 2, 3, 4, 6, 7, 8, 12, 13, 14, 16*

*Findings from Paper 1 indicate that cultural and religious beliefs significantly influence health decisions within the South Asian community. Most of the sample from paper 1 felt that their faith was more powerful than medicine, believing that prayers could enhance their health. While some opted out of medical treatments due to these beliefs, others felt that combining both could lead to the best health outcomes. Paper 2 expands on this notion of faith, proposing that many south Asian people perceive their illness as a predestined and natural consequence of aging. While this was problematic in the sense that it tended to decrease the likelihood of help-seeking behaviours, for some it was positive as they saw illness as a sign from God to improve their lifestyle, and make meaningful change, reclaim independence. Paper 3 reciprocated this positivity showing that faith improved south Asian patients ability to cope with sickness in more positive ways promoting acceptance and resilience. Doctors who shared their faith were effective in maintaining engagement and interest in treatments, suggesting collective approaches that brought together ‘like-minded’ individuals to be effective for this population. Paper 4 builds on this collectivist mentality suggesting collaboration to be important for this demographic going forward. However, this paper noted distinct challenges related to faith showing that Gods such as ‘Bani’ implied healthcare to be unnecessary and so, prayer and faith was prioritised. Due to this many regularly took part in spiritual activities to deal with sickness which on one hand increased acceptance, however discouraged south Asian people from attending health care appointments and medical treatment. While faith can be excellent in helping people cope with sickness, ultimately it was a notable barrier to seeking knowledge and implemented real and effective lifestyle changes. Paper 6 reciprocated this as participants felt that outcomes of sickness were predetermined for them and so, treatment was redundant. To overcome these barriers this paper notes that HCP’s must be more culturally competent, understanding their cultural needs and beliefs in greater detail so that they can build trust and use faith to encourage uptake in healthcare, rather avoiding it. Increased patient involvement was suggested to help the relationship between patient and HCP. Cultural representation helped, with Gujrati-speaking nurses being key to many participants continued interest. Paper 7 however noted the strain on providing such tailored treatment, suggesting personalised cultural advice could be difficult given the diversity of south Asian patients. However, help with confusion and overwhelming information exchange could be a feasible issue to resolve. Paper 8 also noted that help specifically for females was required due to many families’ revolving around the healthcare of the males. Paper 12 builds on this showing south Asian women to be unable to participate in many group treatments e.g., cardiac rehabilitation, due to the mixed gender aspect conflicting with their cultural beliefs. Additionally, south Asian women tended to feel embarrassed by the types of exercise involved in cardiac rehabilitation, and that they did not accommodate their cultural garments. Additional barriers reflected that of previous data, fatalism, alongside traditional events like Ramadan clashing with their classes causing low attendance. Paper 13 embraced these cultural practices holding group sessions on T2D in a gurdwara. Using cultural vocabulary within the name also facilitated attendance as it was relatable for them, with many participants being particularly moved by the speaker being south Asian. Finally, as the gurdwara is associated with positivity such as health, shelter, equality, the venue was felt to be ideal in terms of increasing accessibility and motivation to attend. Paper 14 also found the success of a healthy dance group to be attributed a culturally appropriate venue, highlighting the need for culturally sensitive health and wellness programmes. That said, many barriers persisted. Family members often forbade participation due to cultural reservations about the appropriateness of dancing. Additionally, despite being all women, some attendees were hesitant to remove their headscarves during exercise which often prevented full participation. Some participants also felt self-conscious when wearing the sporty attire required for the types of exercise, as it conflicted with their cultural identity. Some participants also noted embarrassment around subjects such as sexual health, and were resistant to westernised dietary advice. Like paper 13, rehabilitation sessions also tended to clash with prayer times, with safety concerns in high-crime areas also deterring attendance. While transport provision was a key facilitator of participation it sometimes backfired due to inappropriate gestures such as the use of horns, and issues with the taxi driver’s age and sex preventing them from riding alone. Paper 16 showed that these types of religiously sensitive issues to cause significant distress for south Asian people. To ensure retention and patient wellbeing cultural factors must be recognised and adhered to where possible. Equally, acknowledging south Asian patients beliefs brought improved emotional well-being which ultimately aided their physical recovery suggesting further reason to employ cultural modifications.*

*Knowledge and Education: Reciprocal Synthesis of papers 3, 10, 11, 12, 13, 16, 17*

*Findings from paper 3 highlight that knowledge and education are crucial in facilitating access to healthcare for South Asian people. Cardiac rehabilitation is most beneficial when it is culturally relevant, particularly focusing on food and diet. Regular private contact and personalised guidance from HCP’s are also important for this demographic. Paper 10 found that group DVD sessions are useful for South Asian populations. These sessions are most effective when they use simple language and clear visuals. DVD’s that inform people about their medical treatments and explain complex topics help increase trust between healthcare providers and South Asian patients, as well as encourage engagement with the services. The involvement of medical professionals in group interventions is another useful element. Participants in paper 11 valued the knowledge of HCP’s, appreciating the exchange of information between patient and expert. They were generally eager to learn about their health condition and possible treatments, finding the knowledge from classes and HCP’s to be essential post-CVD diagnosis. Community members and relatives played a key role in their participation, as shared positive experiences encouraged them to contact the service. However, paper 12 noted that language barriers persist, to the extent that HCP’s, particularly cardiac rehabilitation nurses, were unrecognised. For South Asian patients to receive the most effective cardiac rehabilitation, it must be culturally sensitive and offer basic, clear education that first introduces the role of the nurses, followed by a simple understanding of the values of cardiac rehabilitation. Paper 13 supports this, showing that clear visual health messages significantly improve community health behaviours and uptake. Additionally, personal stories from community members were transformative, encouraging the sharing of information and the implementation of integral life changes. Paper 16 builds on this by showing that personalised distribution of information is more effective for this demographic. Individualised attention is impactful for South Asian people. Participants noted the need for longer, less rushed appointments and a slower pace of information exchange to ensure understanding. Patients who felt rushed were unlikely to absorb information from HCP’s and instead relied on generic literature, which may not fully address their needs or circumstances. Finally, paper 17 also showed personal experiences and social interactions to considerably contribute South Asian people’s knowledge and education about health. Like the other papers, it found that community influences are key to education and behaviour change, suggesting that community involvement is crucial in this capacity.*

*Accessibility: Reciprocal Synthesis of papers  3, 5, 9, 11, 12*

*Paper 3 highlighted significant transportation issues affecting access to cardiac rehabilitation for south Asian people. Many venues were too far to walk, necessitating a car, or had parking restrictions that made it difficult for patients, especially those from low-income households, due to distance or cost. Additionally, sessions were often scheduled during busy times, conflicting with working hours or prayer times. Paper 5 found that exercise sessions felt inaccessible for people post-heart attack. However, participants valued the use of professional equipment, which made them feel safe to participate, suggesting a need for better communication about these resources. Paper 9 echoed these findings, showing that despite a willingness to join, barriers such as distance, transportation, work conflicts, and long wait times, particularly for those referred through a universal electronic method, hindered engagement and participation. Paper 11 continued this narrative, showing that while some patients overcame transportation barriers due to the importance they placed on their health, those who worked faced challenges with travel distance and limited evening class availability, making sessions completely inaccessible. Finally, Paper 12 also identified health issues, time constraints, and transportation difficulties as barriers to attending cardiac rehabilitation sessions. Those without access to personal transport found public transport difficult or too expensive, indicating a need for therapies to be offered in more accessible local spaces.*

*Medical and Professional Roles: Reciprocal Synthesis of papers 3, 4, 5, 6, 9, 11, 12, 14, 16*

*Findings from paper 3 show HCP’s to be crucial in the uptake of south Asian people in group treatment like cardiac rehabilitation. HCP’s offer a form of support and reassurance to patients through education; increasing their confidence to make reasonable accommodations to their lifestyle that improve their health and recovery. This paper highlights the importance of a collaborative relationship, stating that it helps patients to feel pride in their involvement, progress, and reflect on how they have overcome obstacles. This is also suggested to help with retention. Papers 4 and 5 also found HCP’s to bring a sense of confidence to south Asian patients. Drawing on the same sample, these studies found it was important to participants to have access to professional monitoring of their vitals i.e., heart rates and blood pressure to feel safe within the cardiac rehabilitation appointment. This component not only brought them a sense of personalisation, but helped them learn about their body and how it reacts to certain exercises. After this learning participants tended to feel a state of empowerment, feeling more able to exercise alone and more frequently; improving both their physical and psychological wellbeing. This need for personalised care and attention from HCP’s was also echoed in paper 6 as south Asian participants stated how a Gujarati-speaking Nurse had bridged language gaps and helped them feel represented within the healthcare through sharing the same culture. Additionally, south Asian patients felt that there to be an ongoing need for personalised care including clearer explanations of health conditions. Paper 9 continues this narrative of HCP influence. Ultimately south Asian participants tended to feel that attending group therapies like cardiac rehabilitation were optional. That said, participants noted that if held responsible by their doctor, they would attend. Considering this, stressing the importance of cardiac rehabilitation through authoritative figures, like doctors, is suggested to increase participation. Paper 11 builds on these findings showing south Asian culture to highly respect the role and thoughts of doctors; a potentially key component to increasing uptake in programmes like cardiac rehabilitation. HCP’s are particularly effective for south Asian people when they make the patient feel valued and supported, and as a byproduct empowered to attend sessions like cardiac rehabilitation. Given the findings, the gravity of HCP’s on south Asian patients attitudes and retention in group programs must be recognised. Paper 12 investigates a more negative view of HCP’s, showing how stigma and alienation can occur towards patients, particularly those who show atypical presentations of health conditions. Patients who experienced this reported feeling withdrawn as they had to ‘prove’ their diagnosis, relying on blood tests to support their claims. Going forward HCP’s must be mindful of potential power complexes that may exist between them and patients, being careful to not discourage patients from attending healthcare appointments. This is particularly important for those from underserved backgrounds e.g., south Asian populations. Paper 14 however focused more on the positive aspects that HCP’s can bring. Presence of qualifications or proof of expertise tended to provide south Asian patients a sense of trust, safety, and confidence; again, displaying how the knowledge and authority assumed with the nature of their position can increase participation in south Asian cultures. Considering this, it is clear that HCP’s can be very impactful on south Asian patients, however their attitudes determine if this influence is positive or negative. Finally, paper 16 showed discomfort to be a key reason for south Asian people not returning to group interventions. Lack of choice or autonomy was identified as a significant barrier to participation for which individualised, tailored treatment from HCP’s could help prevent.*

References

References

[1]. Dilla, D., Ian, J., Martin, J., Michelle, H., & Felicity, A. (2020). “I don’t do it for myself, I do it for them”: A grounded theory study of South Asians’ experiences of making lifestyle change after myocardial infarction. Journal of Clinical Nursing, 29(19-20), 3687-3700.

[2]. Darr, A., Astin, F., & Atkin, K. (2008). Causal attributions, lifestyle change, and coronary heart disease: illness beliefs of patients of South Asian and European origin living in the United Kingdom. Heart & Lung, 37(2), 91-104.

[3]. Galdas, P. M., & Kang, H. B. K. (2010). Punjabi Sikh patients’ cardiac rehabilitation experiences following myocardial infarction: a qualitative analysis. Journal of clinical nursing, 19(21‐22), 3134-3142.

[4]. Galdas, P. M., Oliffe, J. L., Kang, H. B. K., & Kelly, M. T. (2012). Punjabi Sikh Patients’ Perceived Barriers to Engaging in Physical Exercise Following Myocardial Infarction. Public Health Nursing, 29(6), 534-541.

[5]. Galdas, P. M., Oliffe, J. L., Wong, S. T., Ratner, P. A., Johnson, J. L., & Kelly, M. T. (2012). Canadian Punjabi Sikh men’s experiences of lifestyle changes following myocardial infarction: cultural connections. Ethnicity & health, 17(3), 253-266.

[6]. Webster, R. A., Thompson, D. R., & Mayou, R. A. (2002). The experiences and needs of Gujarati Hindu patients and partners in the first month after a myocardial infarction. European Journal of Cardiovascular Nursing, 1(1), 69-76.

[7]. Bandyopadhyay, M. (2021). Gestational diabetes mellitus: a qualitative study of lived experiences of South Asian immigrant women and perspectives of their health care providers in Melbourne, Australia. BMC Pregnancy and Childbirth, 21, 1-12.

[8]. Astin, F., Atkin, K., & Darr, A. (2008). Family support and cardiac rehabilitation: a comparative study of the experiences of South Asian and White-European patients and their carer’s living in the United Kingdom. European Journal of Cardiovascular Nursing, 7(1), 43-51.

[9]. Grewal, K., Leung, Y. W., Safai, P., Stewart, D. E., Anand, S., Gupta, M., ... & Grace, S. L. (2010). Access to cardiac rehabilitation among South-Asian patients by referral method: a qualitative study. Rehabilitation Nursing Journal, 35(3), 106-112.

[10]. Patel, N., Stone, M. A., Hadjiconstantinou, M., Hiles, S., Troughton, J., Martin-Stacey, L., ... & Khunti, K. (2015). Using an interactive DVD about type 2 diabetes and insulin therapy in a UK South Asian community and in patient education and healthcare provider training. Patient education and counselling, 98(9), 1123-1130.

[11]. Banerjee, A. T., Grace, S. L., Thomas, S. G., & Faulkner, G. (2010). Cultural factors facilitating cardiac rehabilitation participation among Canadian South Asians: a qualitative study. Heart & Lung, 39(6), 494-503.

[12]. Chauhan, U., Baker, D., Lester, H., & Edwards, R. (2010). Exploring uptake of cardiac rehabilitation in a minority ethnic population in England: a qualitative study. European Journal of Cardiovascular Nursing, 9(1), 68-74.

[13]. Coe, C., & Boardman, S. (2008). From temple to table: an innovative community health and lifestyle intervention aimed at a South Asian community. Ethnicity and Inequalities in Health and Social Care, 1(2), 44-51.

[14]. Visram, S., Crosland, A., Unsworth, J., & Long, S. (2008). Engaging women from South Asian communities in cardiac rehabilitation. International Journal of Therapy and Rehabilitation, 15(7), 298-305.

[15]. Jolly, K., Taylor, R., Lip, G. Y., Greenfield, S., Raftery, J., Mant, J., ... & Stevens, A. (2007). The Birmingham Rehabilitation Uptake Maximisation Study (BRUM). Home-based compared with hospital-based cardiac rehabilitation in a multi-ethnic population: cost-effectiveness and patient adherence. Health Technology Assessment (Winchester, England), 11(35), 1-118.

[16]. Jolly, K., Greenfield, S. M., & Hare, R. (2004). Attendance of ethnic minority patients in cardiac rehabilitation. Journal of Cardiopulmonary Rehabilitation and Prevention, 24(5), 308-312.

[17]. Jones, M., Jolly, K., Raftery, J., Lip, G. Y., & Greenfield, S. (2007). ‘DNA ‘may not mean ‘did not participate’: a qualitative study of reasons for non-adherence at home-and centre-based cardiac rehabilitation. Family practice, 24(4), 343-357.
