# Supplementary material for: Understanding barriers and facilitators to education and rehabilitation interventions for South Asian people with long-term conditions: a systematic review and meta-ethnography
Source: BMJ Open. 2026 Jan 13;16(1):e106694. doi: 10.1136/bmjopen-2025-106694 (PMC12815045; doi:10.1136/bmjopen-2025-106694)
Supplement: online supplemental file 5 [file bmjopen-16-1-s005.docx]

**Box 1: Supporting Quotes on Cultural Barriers**

*“…if there are religious restrictions for example you ask me to go an exercise class where there is the mix (of men and women), then I would not go, even if I really need to go”* [1]*.*

*“[I] wouldn’t fit in there”* [2]*.*

*“Here, you don’t have any companions”*[3]*.*

*“The food we eat is different to what they eat here, and what we are told is that all this “English” food is good and “ours is not”. For us following this is hard, because people here eat steamed vegetables and meat and all those sorts of things. But we are used to eating different kinds of things. All this is too difficult you know; we are already going through the stress of having GDM, and then we have to now change our food habits as well...”*[4]*.*

*“All they know is that ‘we eat rice and curries’, that is all”* [4]*.*

*“We don't talk a lot about it she gets upset and sometimes she says oh I don't know what's happened to me she starts crying so we try not to mention any of it in front of her or something...if there's like something that's gonna make her better I read something and I tell her that oh that's gonna make you better, you better try doing that and stuff”*[5].

*“At the beginning we thought that she can't go on the bus, she can't walk up here, she can't go here and my husband would take her ... in the car he used to take her here and there, even if she had to get the shopping we had to take her because we used to think we had to be with her, she can't go by herself ... She was like [thinking]... I'm getting locked up inside and are they always going to treat me like that?”*[5]*.*

*“My husband's always saying “Oh ... when you're cooking take a bit out for yourself. You know, if there’s chicken and vegetables, separate it and you have it before ... we add the oil to it”. But it’s me myself. I think, “Oh what’s the point? I’ll just eat whatever everyone else is eating”. I just decide to eat less of what they’re eating”*[5]*.*

*“I am always telling her but whether she understands is another matter and it is of benefit to her. If her health is good then I will benefit also. For her it is a matter of understanding. If I keep on about it, then she sees it as a bad thing”*[5]*.*

*“…their families, if they don’t feel that they are going to be comfortable there, they object as well”*[6]*.*

*“a relief dietitian had done [an educational talk] and didn’t know much about the Asian diet and suggested dhal, and that is seen as what poor people eat and a bit of a slur that she was saying that they should eat that”*[6]*.*

**Box 2: Supporting Quotes on Cultural Facilitators**

*“...when in crisis we would first rely on our family first because Asians are family oriented”*[1]*.*

*“If it wasn't for my family, I wouldn't be able to say no to what I was drinking and eating. In the programme I attended they said it's your goal this is the target, and I come home sit down with others, even my deaf mom, bless her soul, she will come and sit around and I will go through what I learned and we make it our target. Then it's easy”*[1]*.*

*“It was good in hospital because there was a Gujarati nurse, so if we had any problems about food or anything If there is one of ours we can say what we mean, and it makes a big difference”*[7]*.*

*“…if there’s a booklet or something for people from the Indian sub-continent”*[4]*.*

*“She was like quite surprised and quite concerned, because in India I mean you don’t really see, I mean women being pregnant and put on insulin. It was like very stressful for me after my diagnosis like with no family support and working full-time”*[4]*.*

*“You really related to her because she was from an Asian background. It was powerful”*[8]*.*

*“We are used to receiving help from family; it's our duty to help”*[4]*.*

*“My daughter keeps books with her, I think she keeps all of the books with her, she reads them regularly, she says to me mum if I'm not here...she doesn't live here, she lives in another house, she tells me if anything happens to daddy then...it tells you in the book what to do and she explains to me what to do and she says do this, I'll explain it to you... she's always explaining things to us...”*[5]*.*

*“I have spoken to some of my friends who had a heart attack, and they always attended rehab and told me lots of positive things about how it helped them to lead a better life and what to eat and not to eat”*[9]*.*

*“Yes, they [i.e., family] are very important. My wife forced me to come here and she made the appointment, and she comes with me to the lectures”*[9]*.*

*“[my] youngest son said, ‘Dad, you get ill, sometimes you get this, sometimes you get that pain so give up your cigarettes. Who else will I have if I don’t have you?’ because I do not have a real brother of my own, I am the only one”*[8]*.*

**Box 3: Supporting Quotes on Faith Barriers**

*“…it is up to God what he does to us, first he makes your hair white, then he gives you eye problems then knee problems. These are all warning signs from Him above, it is like God telling us what is to come”*[10]*.*

*“Whatever pain you were meant to have you’re going to have, no matter how much you take care of yourself. Whatever is in your kismet, whatever is written, that is what is going to happen”*[2]*.*

*“…sometimes I think…that if I don’t take medicine, if I only do prayer, prayer, prayer, I can become better, that is also coming in my mind, now… for the last days, you know… ‘Why should I go for medicine?’’ It is written ‘‘Sarab rog ka aukhad naam’ [the recitation of God’s name cures all diseases]”*[2]*.*

*“If we are called, we have to go. We have to finish what is written from upstairs and we have to go through what is written for us”*[7]*.*

*“You cannot trust life, it is in nature’s hands. We have to suffer what is written down in our individual fate”*[7]*.*

*“With God’s mercy I’m happy in every way. It’s in God’s hands now”*[7]*.*

*“If God wishes it, then I will get better, otherwise it will be just like this”*[7]*.*

*‘I don't understand why I had a heart attack, can't say it's because I eat something that's why I had it or I did something that's why I had it. I don't know why, only God knows”*[11]*.*

*“Ghee makes you strong” (Coe & Boardman, 2008)*[8]*.*

*“Look... the children, if they want something they will do it immediately but it’s the older folks who resist change”*[8]*.*

*“I am now 80 years old and it is up to God what he does to us, first he makes your hair white, then he gives you eye problems then knee problems. These are all warning signs from Him above, it is like God telling us what is to come . . . I think [thick blood] is my main problem because my diet is OK, so I believe it was in my fate as well. This is just an excuse, we have all got to go”*[8]*.*

**Box 4: Supporting Quotes on Faith Facilitators**

*“If you accept all of it, then it doesn’t even come into your mind. When Maharaja [the great or high King] is going to give you any dukh [pain or suffering] or take away the dukh, that is not the thing. If someone asks me how I am, I respond by saying, ‘I’m very well, brother’. You shouldn’t panic about these things, you shouldn’t be afraid of death”*[2]*.*

*“It [illness] comes from Allah (God) . . . whatever’s going to happen, but then you should still be careful shouldn’t you...Allah will do whatever he wants to do . . . but Allah also says that you can do things for yourself so you should be aware of the cause of it”*[8]*.*

*“It’s a very good thing, this is the place where people meet each other, have a good chat... about everything, what’s been going on... it’s a good place to talk about these things”*[8]*.*

*“According to our religious teachers, Gurdwara can be used for anything good. Not only for prayers and worship. So anything where people and community would benefit could be undertaken at the Gurdwara. So I think it’s a great place to discuss health issues. It is a great place because it’s easily accessible”*[8]*.*

**Box 5: Supporting Quotes on Communication Barriers**

*"People go with me [to the hospital] who can speak English and they tell me when I have an appointment... I don't go on my own. Whenever I go, I take someone with me. I don't know anything on my own...if nobody is with me then I can't do anything"*[5]*.*

*“... if there’s somebody there [at the gym] to tell me that would be beneficial ... how much English I need, you know, so I can get by with the English I have, I worked in security, I had to speak in English, so little things I understand”*[3]*.*

*“They speak English, we speak Gujarati; poor things, how can they explain? It’s not their fault they speak English. Nobody even told me I’d had a heart attack”*[7]*.*

*“Cardiac rehabilitation nurse, who is that?”*[11]*.*

*“Some of the talks were very informative and I would like to listen to them, but if you insist that I carry out all these exercises I’m not gonna attend...They’re not written as individuals. They’re just written as a bunch and everyone’s gotten the same. Everyone’s got different needs”*[12]*.*

**Box 6: Supporting Quotes on Communication Facilitators**

*“It was quite easy for somebody who doesn’t know about anything, so it was quite easy and a very, very simple way to explain the benefits of the insulin”*[13]*.*

*“Yeah, that was good, so it’s like yeah OK I've got the answer right and you smile, it’s the human reaction and it’s good to have that smiley face and not having a smiley face”*[13]*.*

**Box 7: Supporting Quotes on Patient-Provider Relationship Barriers**

*“It makes my wind (flatus) problem worse... I just don't eat it. Because we pray regularly, we need to be constantly in ablution. Making wind makes us not so. So I did not do what they asked me to do”*[1]*.*

*“Currently everything is a matter of trial and error for us. If we can get a bit more personalised care, it would be very helpful”*[4]*.*

*“[Healthcare providers] were not too emphatic about it...They do not, you know, emphasize on how important it is. It is just like any other instruction set that they give you...the only suggestion that I have is that they could make it kind of, you know, a little more emphatic and highlight the benefits of the program”*[14]*.*

*“The only time I heard about [CR] was through the letter, which I received probably a week to 10 days ago”*[14]*.*

*“If they were to call me today, I would go. This is the proper time to do it, not after 4 months”*[14]*.*

*“Well, I was told about it by the other patients who had the same bypass surgery that you can go to rehab. So I asked my doctor and he recommended me to rehab”*[14]*.*

*“I think why other people are not coming is all in the hands of the family doctor. They have a role to play and give a little pressure. All South Asians listen to doctors and respect them. If they take an extra 5-10 minutes to say we should go to CR, it would be good”*[9]*.*

*“The first lady came she was a Nurse, I think, she was not the right person, she made joke out of me. Like I was a young man, what was wrong with me?”*[11]*.*

*“She was trying to force you know, the things they wanted me to do, whether you’re able to do them or whether you can’t cope with them”*[12]*.*

*“Some of the talks were very informative and I would like to listen to them, but if you insist that I carry out all these exercises I’m not gonna attend...They’re not written as individuals. They’re just written as a bunch and everyone’s gotten the same. Everyone’s got different needs”*[12}*.*

*“I would rather die than do that, but I dunno, at that time and the situation I had to go along with it, but I wasn’t very happy, to say the least”*[12]*.*

*“I spoke to the surgeon there and he, you know, the other staff and they insisted that, you know I got to have this haircut, although I haven’t got a lot of hair, but they say for this much piece has got to be clean. I have a written article on this somebody in Amritsar in the Punjab...and they have carried out a lot of surgeries...and they say, you know, if you don’t cut the hair actually it, helps the healing the wound a lot quicker. ...because if somebody is Sikh ... it helps them you know to recover more quicker if they don’t cut their hair... but they insisted I had it, ... I would rather die than do that, but I dunno, at that time and the situation I had to go along with it, but I wasn’t very happy, to say the least”*[12]*.*

**Box 8: Supporting Quotes on Patient-Provider Relationship Facilitators**

*“…when we go to the exercise classes ... they check your blood pressure, the heart rate monitor watch is connected, plus they check your oxygen ... there are 2 bikes and 4 treadmills ... there are 6 to 8 people there, and every minute the physiotherapists, rehabilitation nurse, and the other nurses check us, they are all very attentive. The big thing that I have noticed is that they give us time, they don’t hurry us, they want each of us to know the information and they tell us enthusiastically what we need* t*o know, and then we can happily ask them questions, because the environment is very friendly”*[3]*.*

*“…if you just go to the gym, you don’t know what exercise they’ll give you, here [CR] they’ll give you guidance”*[3]*.*

*“The program is good because they give me training so I am safe. If I do something on my own at home and something happens, I don’t know what will happen to me. So the fact it is medically supervised. I feel comfortable doing it under the supervision of TRI”*[9]*.*

*“Yes, he’s [i.e., the cardiologist] happy to see my progress and asks me about the program. Doctors are respected in our South Asian culture and we abide them, and there is reason why they tell us to do things and we should follow it”*[9]*.*

*“My doctor friends, I talked to 4 doctors, and they said bypass is very good because you are very healthy now and success rate is very high”*[11]*.*

*“Then there was more telling how the medicines ... This was quite good. I liked it. ... Yes, it was very, very helpful. Which medicine they give you, and what they do. And you can’t take this medicine with that, its worse for you or harming you”*[12]*.*

**Box 9: Additional Quotes on Accessibility**

*“The time, sometimes it’s hard for somebody to drop me off and pick me up. That’s really hard, but other than that it’s totally fine, it’s just the timing that’s a bit off”*[2]*.*

*“No parking is the main problem there. You know and if you get there a bit early you can park a bit farther away and, you know, I try to park on the streets because then I can get a walk in, its farther”*[2]*.*

*“Ten is a long time, and I might have to take time off, but because of their evening classes I am coming. If I had to take time off from work to come here in the mornings, I would stop coming”*[9}

*“It is quite far from my place, but I have a car so I drive over, which makes it a bit easier. I know it’s important I come here for my health, so the distance doesn’t matter” (Banerjee et al., 2010)*[9]*.*

*“I mean, I would not have minded (going) but I had to go to Darwen [local town] and I couldn't make it and you can't afford to take a taxi every time, it is expensive. Most afternoons I work so it was difficult”*[11]*.*

*“I couldn't go to because I would have to use the bus and change twice on my own. It would just be difficult in case something happened. I do get a lot of pains in my legs”*[11]*.*

References

[1]. Dilla, D., Ian, J., Martin, J., Michelle, H., & Felicity, A. (2020). “I don’t do it for myself, I do it for them”: A grounded theory study of South Asians’ experiences of making lifestyle change after myocardial infarction. Journal of Clinical Nursing, 29(19-20), 3687-3700.

[2]. Galdas, P. M., & Kang, H. B. K. (2010). Punjabi Sikh patients’ cardiac rehabilitation experiences following myocardial infarction: a qualitative analysis. Journal of clinical nursing, 19(21‐22), 3134-3142.

[3]. Galdas, P. M., Oliffe, J. L., Wong, S. T., Ratner, P. A., Johnson, J. L., & Kelly, M. T. (2012). Canadian Punjabi Sikh men’s experiences of lifestyle changes following myocardial infarction: cultural connections. Ethnicity & health, 17(3), 253-266.

[4]. Bandyopadhyay, M. (2021). Gestational diabetes mellitus: a qualitative study of lived experiences of South Asian immigrant women and perspectives of their health care providers in Melbourne, Australia. BMC Pregnancy and Childbirth, 21, 1-12.

[5]. Astin, F., Atkin, K., & Darr, A. (2008). Family support and cardiac rehabilitation: a comparative study of the experiences of South Asian and White-European patients and their carer’s living in the United Kingdom. European Journal of Cardiovascular Nursing, 7(1), 43-51.

[6]. Visram, S., Crosland, A., Unsworth, J., & Long, S. (2008). Engaging women from South Asian communities in cardiac rehabilitation. International Journal of Therapy and Rehabilitation, 15(7), 298-305.

[7]. Webster, R. A., Thompson, D. R., & Mayou, R. A. (2002). The experiences and needs of Gujarati Hindu patients and partners in the first month after a myocardial infarction. European Journal of Cardiovascular Nursing, 1(1), 69-76

[8]. Coe, C., & Boardman, S. (2008). From temple to table: an innovative community health and lifestyle intervention aimed at a South Asian community. Ethnicity and Inequalities in Health and Social Care, 1(2), 44-51.

[9]. Banerjee, A. T., Grace, S. L., Thomas, S. G., & Faulkner, G. (2010). Cultural factors facilitating cardiac rehabilitation participation among Canadian South Asians: a qualitative study. Heart & Lung, 39(6), 494-503.

[10]. Darr, A., Astin, F., & Atkin, K. (2008). Causal attributions, lifestyle change, and coronary heart disease: illness beliefs of patients of South Asian and European origin living in the United Kingdom. Heart & Lung, 37(2), 91-104.

[11]. Chauhan, U., Baker, D., Lester, H., & Edwards, R. (2010). Exploring uptake of cardiac rehabilitation in a minority ethnic population in England: a qualitative study. European Journal of Cardiovascular Nursing, 9(1), 68-74.

[12]. Jolly, K., Greenfield, S. M., & Hare, R. (2004). Attendance of ethnic minority patients in cardiac rehabilitation. Journal of Cardiopulmonary Rehabilitation and Prevention, 24(5), 308-312.

[13]. Patel, N., Stone, M. A., Hadjiconstantinou, M., Hiles, S., Troughton, J., Martin-Stacey, L., ... & Khunti, K. (2015). Using an interactive DVD about type 2 diabetes and insulin therapy in a UK South Asian community and in patient education and healthcare provider training. Patient education and counselling, 98(9), 1123-1130.

[14]. Grewal, K., Leung, Y. W., Safai, P., Stewart, D. E., Anand, S., Gupta, M., ... & Grace, S. L. (2010). Access to cardiac rehabilitation among South-Asian patients by referral method: a qualitative study. Rehabilitation Nursing Journal, 35(3), 106-112.
