# Supplementary material for: Understanding barriers and facilitators to education and rehabilitation interventions for South Asian people with long-term conditions: a systematic review and meta-ethnography
Source: BMJ Open. 2026 Jan 13;16(1):e106694. doi: 10.1136/bmjopen-2025-106694 (PMC12815045; doi:10.1136/bmjopen-2025-106694)
Supplement: online supplemental file 7 [file bmjopen-16-1-s007.docx]

**Table 2:** Article Characteristics

| **Author & Year of Publication** | **Country** | **Population** | **Health Condition** | **Type of Group Treatment** | **Data Collection** | **Analysis** | **Research Questions/Aims** | **Key Findings** |
| --- | --- | --- | --- | --- | --- | --- | --- | --- |
|  |  |  |  |  |  |  |  |  |
| Astin et al., 2008  [1] | UK | 20 White-European and **45 South Asian patients** aged over 30 years, with a diagnosis of unstable angina (32%), MI (42%) or for CABG surgery (26%). | CHD / MI | Cardiac Rehabilitation | Semi-structured interviews, between 60 and 90 minutes. | Framework Analysis | The aim of this exploratory qualitative study was to explore the nature of family support available to a sample of UK South Asian and White-European cardiac patients and to highlight similarities and differences between these groups with regard to cardiac rehabilitation and lifestyle modification. | Main themes: Advice and information provision, family support and burden, dietary change, and exercise regimes.  Cultural and Ethnic differences: - Patients and their families exhibited both cultural differences and similarities, regardless of ethnicity. - These patterns may reflect general recovery characteristics after a cardiac event.  Health Professionals’ Role: Health professionals should cultivate a cultural understanding to engage with diversity. - Not all difficulties in accessing services can be attributed solely to ethnic background.  Improving Support: - Despite this, enhancing services would still benefit South Asian populations, however the challenge lies in discerning when ethnicity impacts service support and when it does not. |
| Bandyopadhyay, 2021  [2] | Australia | 21 health care providers and **23 South Asian women**: 13 Indian, 5 Pakistani, 3 Sri Lankan, 1 Indo-Fijian, 1 2nd Gen Indian Australian. | Gestational Diabetes | Educational Class | Semi-structured interviews, on-to-one, and observation in both group settings and at an individual level. | Constant Comparative Methods | To gain a better understanding of the lived experiences of South Asian women and their experiences of self-management and their health care providers’ perspectives of treatment strategies. | Clinical Practice Challenges: - The current clinical practice falls short in terms of self-management behaviour, a crucial aspect of gestational diabetes management. - Health care providers struggle to encourage South Asian women to self-manage their blood glucose levels through lifestyle modification.  Cultural Awareness Needed: - Health care providers should recognise diverse patient cultures. - The current generic approach fails to engage and meet the needs of immigrant and ethnic women.  Future Strategies: - Develop, evaluate, and disseminate appropriate diet plans, models, and resources for South Asian women diagnosed with gestational diabetes. |
| Banerjee et al., 2010  [3] | Canada | 16 Canadian South Asian participants in a 12-month CR program | Myocardial Infarction | Cardiac Rehabilitation | Researchers conducted semi-structured one-on-one interviews lasting approximately 45 minutes. The interview guide, based on the PRECEDE model, aimed to facilitate discussions about participants’ perceptions, expectations, and actual experiences with a CR program. Topics covered included factors influencing participation decisions, program likes and dislikes, and suggestions for improvements. | NVivo: Thematic analysis | To explore the potential cultural factors that facilitate participation in on-site CR sessions among South Asian patients with cardiac disease living in Canada. | Obstacles to Participation: - Some obstacles (e.g., age, disease severity, diagnosis, sex, and educational attainment) are permanent and cannot be changed thus, it is crucial to focus the factors that can be changed, such as maximising referral rates.  South Asians and CR: - South Asians diagnosed with CVD do have the capacity to utilise CR services, with facilitators for CR participation among South Asians existing at both universal and cultural levels. - Data on facilitators to CR attendance and adherence should inform the planning and implementation of appropriate CR services  Role of Physicians:  - Physicians play a crucial role in referring South Asian patients to CR. - Physicians should emphasise the benefits of CR to South Asian patients during the referral process, assuring south Asian patients that they will receive medical supervision during CR. - Regular follow-up during clinic visits is also essential to maintain attendance.  Family and Community Support:  - Informing family and community members about positive CR experiences and involving them in educational sessions helps maintain lifestyle changes.  Cultural Considerations: Acknowledge cultural preferences while providing CR services. Patient Education: Understanding cultural factors is crucial, but avoid overgeneralizing patient characteristics based on ethnicity |
| Chauhan et al., 2010  [4] | UK | 20 participants (12 Pakistani, 6 Indian and 2 Bangladeshi) eligible for CR | Myocardial Infarction | Cardiac Rehabilitation | Semi-structured interviews, one-to-one. | Framework analysis and Grounded Theory | To explore the experiences of participants following an acute cardiac event; with a specific focus on reasons for the take up of cardiac rehabilitation services. | Access and Experience of CR Services:  - There are problems in both access and experience of CR services for minority ethnic participants.  Communication Difficulties: - Negative experiences related to communication difficulties (e.g., lack of interpreters, atypical symptom presentation) sometimes led to clinical assumptions causing delays or misdiagnoses.  Facilitators:  - Social networks and family (often health professionals) facilitated access to appropriate care.  Addressing Inequality:  - Routine recording of ethnicity, religion, language, and interpreter needs in medical records is crucial to address the needs of specific groups. - This can improve health literacy, which influences CR attendance.  Barriers and Solutions:  - Same-gender classes and flexible class timing can address some barriers mentioned by participants.  Further Research:  - Qualitative work is needed to explore CR nurses’ views on reasons for poor uptake among minority ethnic populations. |
| Coe & Boardman, 2008  [5] | UK | 13 South Asian people with T2D. | T2D | Educational Class | Focus groups | Framework analysis | - To educate this South Asian community on the health risks pertinent to them. - To raise awareness of risks attached to current lifestyle and diet. - To identify changes to improve health outcomes. - To support behaviour change. - To work together as a community. - To use culturally sensitive and acceptable means to achieve these objectives. | Supportive Environment: - Participants felt supported by the initiative, and preferred it over other methods due to the venue and simple visual and narrative formats of health messages.  Success: - Lifestyle changes were achieved at both individual and household levels. - There was also raised awareness, understanding, satisfaction and engagement.  Transferability: - Elements of the Apnee Sehat project create a toolbox that has the potential for transfer to other communities in the UK.  Culture Specific Needs: - South Asian populations have specific healthcare needs.  - Current healthcare delivery structures are not accommodative or appropriate for this community. - Community participation is helpful in instigating and sustaining change, the Gurdwara/places of worship are deemed a natural home for a health and lifestyle intervention. |
| Darr et al., 2008  [6] | UK | 65 people (**20 Pakistani-Muslim, 13 Indian-Hindu**, **12 Indian-Sikh,** and 20 Europeans) who were admitted to one of three UK sites within the previous year with unstable angina or myocardial infarction, or to undergo coronary artery bypass surgery. | CHD / MI | Cardiac Rehabilitation | Semi-structured interviews | Framework analysis | To examine and compare the illness beliefs of South Asian and European patients with CHD about causal attributions and lifestyle change. | Misconceptions and Lack of Understanding:  - Across ethnic groups, there are misconceptions about the causes of CHD and a lack of understanding regarding appropriate lifestyle changes.  Tailoring Information and Advice:  - Health care professionals should better tailor information and advice related to cardiac rehabilitation to the specific needs, beliefs, and circumstances of CHD patients, regardless of their ethnicity.  Similar Concerns and Needs:  - South Asian and European patients with CHD share similar concerns, worries, and needs.  - However, it is essential to recognise that not every problem can be attributed solely to ethnic background. - Socioeconomic status, age, and gender are as important as ethnicity in understanding health and social care needs. Policy guidelines should consider these factors alongside ethnicity.  Challenges in Providing Care:  - Health care providers struggle to offer accessible and appropriate care to ethnic minority populations due to socioeconomic disadvantage and social exclusion.  Cultural Competence:  - Professionals need cultural awareness and sensitivity to engage with diversity. Training should address cultural and religious beliefs, valuing clients as individuals within their communities. Promoting Culturally Sensitive Care: Further research is needed to support CHD patients from diverse ethnic backgrounds. |
| Dilla et al., 2020  [7] | UK | 14 south Asian people, one-month post-diagnosis of myocardial infarction: 4 Pakistani, 6 Indian, 2 Bangladeshi, 1 Kashmiri, 1 Punjab. | Myocardial Infarction | Cardiac Rehabilitation | Longitudinal, face-to-face, semi- structured in-depth interviews | Constructivist grounded theory design. | To explore South Asians’ experience of choosing and prioritising lifestyle changes during their recovery from first myocardial infarction. | Family Influence:  - South Asians often prioritise family needs over their own health. Family plays a significant role in supporting recovery, but this can sometimes cause conflict.  Beliefs and Norms: - Religious and health beliefs impact decision-making. Participants conform to these beliefs, which may influence their lifestyle choices.  Desire for Harmony: - Participants seek “harmony” in their lives, making decisions based on maintaining balance rather than solely addressing individual health needs.  Shared Priorities:  - Instead of focusing solely on individual self-efficacy, a new model of “shared priorities” should be applied in CR programs for South Asians. This approach considers cultural competence and aligns lifestyle advice with family goals and religious beliefs.  Complexities: - Health experiences are shaped not only by biographical factors but also by broader social and cultural complexities. Understanding these nuances is crucial for successful interventions across ethnic groups. |
| Galdas and Kang, 2010  [8] | Canada | 15 Punjabi Sikh patients post myocardial infarction attending a cardiac rehabilitation programme | Myocardial Infarction | Cardiac Rehabilitation | Narrative interviews | Thematic analysis, using grounded theory methods of coding and constant comparative analysis | To explore the cardiac rehabilitation experiences of Punjabi Sikh patients, post myocardial infarction. | Culturally Relevant Rehabilitation Advice: - It is crucial to offer practical, culturally relevant diet and lifestyle advice to ensure that patients understand and can implement necessary modifications effectively.  Ongoing Dialogue and Support:  - Providing time for ongoing dialogue with healthcare professionals and peers is essential. This support helps patients adhere to rehabilitation recommendations and make the necessary lifestyle adjustments.  Avoiding Generalisations:  - Research and practice must recognise that religious beliefs impact people differently. Assumptions about how religious beliefs may affect adherence to CR must be avoided.  Prominent Features of Patients’ Experiences:  - Understanding the unique experiences of Punjabi Sikh patients attending CR is vital. This awareness informs tailored interventions which are necessary for ethnic minorities.   Further Research Needed:  - To enhance CR provision, additional research is required. Exploring themes in other settings and with different South Asian religious groups will contribute to this field. |
| Galdas et al., 2012  [9] | Canada | 27 Canadian Punjabi Sikh men post-MI  **15 participants were recruited from attendees of a weekly hospital-based cardiac rehabilitation** programme for post-MI patients in the lower mainland of BC, Canada. The cardiac programme offered health and lifestyle-oriented education and risk reduction using classes, workshops and individual counselling sessions. | Myocardial Infarction | Cardiac Rehabilitation **(only some of the data covers this).** | Semi-structured interviews | Constant comparative methods | To describe how culture underlies Canadian Punjabi Sikh men’s experiences of adopting lifestyle changes following MI. | Cultural Influence: - Culture significantly influenced Canadian Punjabi Sikh men’s experiences in adopting lifestyle-related changes post-MI. Their cultural practices played a role in shaping their behaviours and choices.  Family and Community Contexts:  - The tasks related to self-care, rehabilitation, and lifestyle changes were deeply embedded in the family and community contexts of these men. Collectivist bonds and customs influenced their decisions.  Balancing Cultural and Individual Needs:  - Canadian Punjabi Sikh men faced challenges in balancing the demands of collectivist cultural and spiritual bonds with the individual lifestyle changes required for post-MI recovery.  Implications for Healthcare Providers:  - Healthcare providers should consider the influence of collectivist cultures when planning and delivering rehabilitation advice to Canadian Punjabi Sikh men post-MI. Culturally appropriate heart health and rehabilitation messages can better support their practices |
| Galdas et al., 2012  [10] | Canada | 15 participants (10 men, 5 women) living in British Columbia, Canada, born in the Punjab region of north India, who had a MI within 6 months of their interview. Ages ranged from 48 to 80 years old. | Myocardial Infarction | Cardiac Rehabilitation | Face-to-face interview | An interpretive thematic approach involving constant comparison (Attride-Stirling, 2001; Strauss & Corbin, 1998) and NVivo | To describe Punjabi Sikh patients perceived barriers to engaging in physical exercise following myocardial infarction. | Determining Safe Exertion Levels:  - Participants faced challenges in independently determining safe levels of physical exertion. Uncertainty about what constituted safe exercise was a recurring barrier.  Satisfaction with Cardiac Rehabilitation Education: - Participants appreciated professional guidance from CR programs regarding exercise and lifestyle changes.  Fatigue and Weakness: - Participants tended to feel weak, invasive procedures like angioplasty or bypass surgery, contributed to cautious exercise behaviour. While surgeries are helpful they created barriers to exercise.  Preference for Informal Exercise:  - Most participants preferred informal activities like brisk walking. Walking outdoors was highly valued for its health benefits and social aspects.  Migration-Related Challenges:  - Participants who migrated to Canada as adults experienced changes in social networks, impacting their opportunities for physical activity. |
| Grewal et al., 2010  [11] | Canada | 16 South-Asian people with an ACS diagnosis (15 male, 1 female) with a mean age of 62 years old. 11 were born in India, 2 from Bangladesh, 2 from Sri Lanka, and 1 person from Uganda. | Myocardial Infarction | Cardiac Rehabilitation | Semi-structured interviews | NVivo: Interpretive descriptive analysis | To qualitatively explore whether CR referral knowledge and access varied among South-Asian patients. | Liaison Referral Category: - Discussions about CR in the hospital often involved nurses or physiotherapists rather than physicians. - Not all patients in this category were referred to CR, highlighting variability.  Referral Methods: - Some patients received English-language documents (letters or pamphlets) about CR while in the hospital, but never had the opportunity to discuss CR with healthcare providers in person. For some this was the only way they knew about CR. - Healthcare providers treated CR instructions similarly to other instructions and did no emphasise the importance.  - Participants tended to hear about CR through their friends and then must ask their doctor for it. Some had prior knowledge from previous rehabilitation programs. - Some patients didn’t enrol in CR centres due to lack of information. - Many patients who hadn’t attended CR were unaware of the full range of services offered. - Some associated CR primarily with exercise. - Patients referred through automatic methods expressed uncertainty about CR benefits.  Personal Autonomy: - Enrolling in CR was considered a personal choice. - External factors played a role, but the final decision was perceived as their own.  Reasons for Not Attending CR: - Some patients believed they could exercise at home or in a gym, deeming CR unnecessary. - A participant mentioned their doctor’s advice: “Doesn’t need, up to you.” Suggesting they felt it was not a requirement. - Some participants felt they had sufficient information to manage and exercise independently.  Barriers to Attendance: - Barriers included program distance, transportation, work conflicts, and wait times. - Wait times were a concern primarily for those referred via universal electronic methods. |
|  |  |  |  |  |  |  |  |  |
| Jolly et al., 2004  [12] | UK | **34 South Asian patients** (2 West Indian, 22 Indian, 1 Gujerat, 1 Subcontinent, 1 Bangladeshi, 1 Kenyan, 4 Pakistani, 2 from non-south Asian countries) and 7 African Caribbean patients, who had experienced a cardiac event (myocardial infarction, coronary artery bypass grafting, percutaneous transluminal coronary angioplasty, or valve replacement) and had been referred for cardiac rehabilitation. | Myocardial Infarction | Cardiac Rehabilitation | Semi-structured interviews | Grounded Theory analysis | To explore ethnic minorities experiences of heart disease and their beliefs about cardiac rehabilitation. | Reasons for Nonattendance: - Language Barrier: Some participants couldn’t speak English. - Shyness and Reluctance to Chat: Others felt very shy. Another participant didn’t want to talk or chat about anything. - Transportation Challenges: One participant found it hard to get to the hospital and back. Some participants had sons who were too busy, and were forbidden from attending alone.  - Perceived Age Limitation: One participant believed they were too old for exercise.  Reasons for Non-Completion: - Participants who didn’t see health improvements dropped out. - Some felt forced to do exercises they couldn’t cope with. Others found the program too troublesome.  Preferences: - Home Program: Some preferred exercising at home for comfort. - Hospital Program: Others appreciated the structured time and professional observation. - Negative Views: Some disliked the uniform approach and lack of mental support in the hospital program Satisfaction with Care Received: - Participant P9 (Female, non-adhered) found everyone helpful. - Participant P21 (Male, attended) felt that everything was done properly.  Perception of Poor Surgical Care: - Participant P6 (Female, refused) believed they were used for practice. - Participant P29 (Female, attended) felt rushed during surgery and questioned the quality of care. - Rushed information: Participant P31 (Male, not completed) received minimal explanation and relied on leaflets for recovery details.  Need for More Information about Prevention: - Participant P6 (Female, declined) wanted more than just answers to questions. - Participant P37 (Male, attended) sought information on avoiding or managing the problem. - Participant P36 (Male, attended) felt professionals were too busy to provide sufficient information.  Religious Insensitivity: -Participant P31 (Male, not completed) faced pressure to cut hair for surgery, despite religious beliefs. |
|  |  |  |  |  |  |  |  |  |
| Jolly et al., 2007  [13] | UK | 58 MI patients who had, or had not, had CR. **(9 South Asian: 7 Indian, 2 Pakistani).** | Myocardial Infarction / Coronary Revascularisation | Cardiac Rehabilitation | Interviews (n=32) and focus groups (n=26) | Charting | **Positive Experiences of CR:** - Patients who participated in CR expressed positive feelings about their involvement.  - Those who attended educational sessions found the medication information particularly beneficial.   **Life Changes:** - After experiencing a cardiac event, all patients were aware of lifestyle changes recommended to enhance their health and reduce the risk of further heart disease, even if they lacked motivation to implement these changes.  - Many reported making adjustments, especially related to smoking and diet. | Positive Experiences of CR: - Patients who participated in CR expressed positive feelings about their involvement.  - Those who attended educational sessions found the medication information particularly beneficial.   Life Changes: - After experiencing a cardiac event, all patients were aware of lifestyle changes recommended to enhance their health and reduce the risk of further heart disease, even if they lacked motivation to implement these changes.  - Many reported making adjustments, especially related to smoking and diet.   Third Party Support: - Patients’ partners and/or family members played a crucial role in supporting positive lifestyle modifications. - Asian patients were motivated when observing dietary changes within their friends and community. |
| Jones et al., 2007  [14] | UK | 49 patients participating in the Birmingham Rehabilitation Uptake Maximisation Study. **9 people from south Asian background: 7 Indian, 2 Pakistani).** | Myocardial Infarction | Cardiac Rehabilitation | Semi-structured interviews | Charting | To explore patients’ reasons for non-participation in or non-adherence to a home- or hospital-based CR programme. | Reasons for Non-Completion of Rehabilitation Programs: - Health issues (e.g., arthritis and ongoing cardiac problems) hindered exercise participation. - Some patients had caregiving responsibilities and couldn’t leave their partners for extended periods. - Poor communication from the hospital was noted by south Asian patients specifically. - Lack of motivation to exercise was the primary reason for non-adherence, especially among women. - Domestic duties in women and ill health in ethnic minority patients also contributed. - Some patients who didn’t attend or adhere to their CR program participated in rehabilitative activities through other means.  Positive Aspects and Lifestyle Changes: - Despite non-adherence, many patients found certain aspects of their cardiac rehabilitation program helpful. However social characteristics, individual patient needs, and program location should be considered in designing CR programs to maximise participation. |
| Patel et al., 2015  [15] | UK | 45 South Asian people with T2D | T2D | Educational Class (DVD) | Qualitative questionnaires | Descriptive analysis | To develop and pilot-test the feasibility and effectiveness of an interactive DVD about misconceptions within South Asian communities regarding insulin treatment in type 2 diabetes, for educating patients and community members and training healthcare providers. | - Patients found the DVD more effective than traditional methods (such as books or verbal explanations) because it provided visual information.  - The DVD simplified complex topics, making it easy for patients to understand the benefits of insulin. Seeing positive reactions (like a smiley face) in the video encouraged patients.  - Some South Asian patients were hesitant to commence insulin therapy. One patient attended but declined study participation, fearing insulin use (despite assurances). - The DVD helped change hesitant patients’ minds about insulin by demonstrating how to handle injections. Staff believed the DVD (if available in different South Asian languages) could address reluctance to start insulin. |
| Visram et al., 2008  [16] | UK | 9 women from South Asian communities | Myocardial Infarction | Cardiac Rehabilitation | Focus group | Thematic content approach | To describe experiences and perceptions of cardiac rehabilitation among a sample of women from South Asian communities. | Barriers to Participation: - Individual, cultural, and practical barriers hinder participation in cardiac rehabilitation: lack of confidence, family commitments, language barriers, and cultural misconceptions - some participants felt the clothing needed for some the cardiac exercises was inappropriate and lack cultural sensitivity.  - Particular topics were also deemed culturally insensitive for a mixed audience; for example, sexual relations and dietary advice. - Interpreters tend to be distracting, there is a need for South Asian language classes.  Facilitators: - Format and content appropriateness. - Venue choice: the women’s dance group thrived due to a familiar venue, supportive leaders, and enjoyable activities, while boosting confidence by being with other women. |
| Webster et al., 2002  [17] | UK | 35 Gujarati Hindu MI patients | Myocardial Infarction | Cardiac Rehabilitation | Semi-structured interviews | Constant Comparative Methods | To explore the experiences and needs of Gujarati Hindu patients and their partners in the first month after a myocardial infarction | Cultural Considerations:  - The experiences and healthcare needs of Gujarati Hindu MI patients differ from those of non-Asians. - Effective treatment for Gujarati Hindu patients depends on awareness of language issues, cultural beliefs, behaviours, and social and family circumstances, suggesting a more culturally sensitive cardiac service to be beneficial to this population. - Family support is vital, and tends to be relied on.  Concerns:  - Patients lacked sufficient information and advice. Patients and partners seemed uninformed about their diagnosis and its implications. All leaflets were given in English, and unread, perhaps for more reasons than one e.g., literacy skills.  - Patients struggled with physical activity, and adjusting lifestyle habits was challenging. - Patients had poor expectations of CR. Only half attended because they were not sure what it entailed or were waiting to be formally invited. - Some patients were dissatisfied with their doctors. - A significant belief in fate was observed, as well as negative thoughts about the future. |

References

[1]. Astin, F., Atkin, K., & Darr, A. (2008). Family support and cardiac rehabilitation: a comparative study of the experiences of South Asian and White-European patients and their carer’s living in the United Kingdom. European Journal of Cardiovascular Nursing, 7(1), 43-51.

[2]. Bandyopadhyay, M. (2021). Gestational diabetes mellitus: a qualitative study of lived experiences of South Asian immigrant women and perspectives of their health care providers in Melbourne, Australia. BMC Pregnancy and Childbirth, 21, 1-12.

[3]. Banerjee, A. T., Grace, S. L., Thomas, S. G., & Faulkner, G. (2010). Cultural factors facilitating cardiac rehabilitation participation among Canadian South Asians: a qualitative study. Heart & Lung, 39(6), 494-503.

[4]. Chauhan, U., Baker, D., Lester, H., & Edwards, R. (2010). Exploring uptake of cardiac rehabilitation in a minority ethnic population in England: a qualitative study. European Journal of Cardiovascular Nursing, 9(1), 68-74.

[5]. Coe, C., & Boardman, S. (2008). From temple to table: an innovative community health and lifestyle intervention aimed at a South Asian community. Ethnicity and Inequalities in Health and Social Care, 1(2), 44-51.

[6]. Darr, A., Astin, F., & Atkin, K. (2008). Causal attributions, lifestyle change, and coronary heart disease: illness beliefs of patients of South Asian and European origin living in the United Kingdom. Heart & Lung, 37(2), 91-104.

[7]. Dilla, D., Ian, J., Martin, J., Michelle, H., & Felicity, A. (2020). “I don’t do it for myself, I do it for them”: A grounded theory study of South Asians’ experiences of making lifestyle change after myocardial infarction. Journal of Clinical Nursing, 29(19-20), 3687-3700.

[8]. Galdas, P. M., & Kang, H. B. K. (2010). Punjabi Sikh patients’ cardiac rehabilitation experiences following myocardial infarction: a qualitative analysis. Journal of clinical nursing, 19(21‐22), 3134-3142.

[9]. Galdas, P. M., Oliffe, J. L., Kang, H. B. K., & Kelly, M. T. (2012). Punjabi Sikh Patients’ Perceived Barriers to Engaging in Physical Exercise Following Myocardial Infarction. Public Health Nursing, 29(6), 534-541.

[10]. Galdas, P. M., Oliffe, J. L., Wong, S. T., Ratner, P. A., Johnson, J. L., & Kelly, M. T. (2012). Canadian Punjabi Sikh men’s experiences of lifestyle changes following myocardial infarction: cultural connections. Ethnicity & health, 17(3), 253-266.

[11]. Grewal, K., Leung, Y. W., Safai, P., Stewart, D. E., Anand, S., Gupta, M., ... & Grace, S. L. (2010). Access to cardiac rehabilitation among South-Asian patients by referral method: a qualitative study. Rehabilitation Nursing Journal, 35(3), 106-112.

[12]. Jolly, K., Greenfield, S. M., & Hare, R. (2004). Attendance of ethnic minority patients in cardiac rehabilitation. Journal of Cardiopulmonary Rehabilitation and Prevention, 24(5), 308-312.

[13]. Jolly, K., Taylor, R., Lip, G. Y., Greenfield, S., Raftery, J., Mant, J., ... & Stevens, A. (2007). The Birmingham Rehabilitation Uptake Maximisation Study (BRUM). Home-based compared with hospital-based cardiac rehabilitation in a multi-ethnic population: cost-effectiveness and patient adherence. Health Technology Assessment (Winchester, England), 11(35), 1-118.

[14]. Jones, M., Jolly, K., Raftery, J., Lip, G. Y., & Greenfield, S. (2007). ‘DNA ‘may not mean ‘did not participate’: a qualitative study of reasons for non-adherence at home-and centre-based cardiac rehabilitation. Family practice, 24(4), 343-357.

[15]. Patel, N., Stone, M. A., Hadjiconstantinou, M., Hiles, S., Troughton, J., Martin-Stacey, L., ... & Khunti, K. (2015). Using an interactive DVD about type 2 diabetes and insulin therapy in a UK South Asian community and in patient education and healthcare provider training. Patient education and counselling, 98(9), 1123-1130.

[16]. Visram, S., Crosland, A., Unsworth, J., & Long, S. (2008). Engaging women from South Asian communities in cardiac rehabilitation. International Journal of Therapy and Rehabilitation, 15(7), 298-305.

[17]. Webster, R. A., Thompson, D. R., & Mayou, R. A. (2002). The experiences and needs of Gujarati Hindu patients and partners in the first month after a myocardial infarction. European Journal of Cardiovascular Nursing, 1(1), 69-76
