# Supplementary material for: Understanding barriers and facilitators to education and rehabilitation interventions for South Asian people with long-term conditions: a systematic review and meta-ethnography
Source: BMJ Open. 2026 Jan 13;16(1):e106694. doi: 10.1136/bmjopen-2025-106694 (PMC12815045; doi:10.1136/bmjopen-2025-106694)
Supplement: Supplementary file 8 [file bmjopen-2025-106694.reviewer_comments.pdf]

# PEER REVIEW HISTORY

BMJ Open publishes all reviews undertaken for accepted manuscripts. Reviewers are asked to complete a checklist review form and are provided with free text boxes to elaborate on their assessment. These free text comments are reproduced below.

## ARTICLE DETAILS

### Title (Provisional)

Understanding barriers and facilitators to education and rehabilitation interventions for South Asian people with long-term conditions: a systematic review and meta-ethnography

### Authors

Shiel, Emma Victoria; Miah, Jahanara; Chattopadhyay, Tapan; Rauf, Abdur; Dalton, Christopher; Husain, Nusrat; Blakemore, Amy

---

## VERSION 1 - REVIEW

---

| | |
|---|---|
| **Reviewer** | **1** |
| **Name** | **Chong, Mei Sin** |
| **Affiliation** | **National University of Singapore** |
| **Date** | **16-Sep-2025** |
| **COI** | **None** |

---

Thank you for the opportunity to review this manuscript, 'Understanding barriers and facilitators to education and rehabilitation interventions for South Asian people with long-term conditions: a meta-synthesis of qualitative studies', which synthesizes the evidence on the experience of South Asian people when attending group treatment programs.

Abstract:

Page 1, line 35-37 – The sentence is incomplete, '..... barriers and facilitators experienced by...' it is not clear barriers and facilitators in what? Please specify the context of these barriers and facilitators. Are they related to accessing healthcare services?

Page 1, line 48 - The results section mentions about 'cardiovascular disease and diabetes mellitus', but one of keywords include 'Chronic Obstructive Pulmonary Disease (COPD)'. Please clarify this.

Introduction:

Page 3, line 13-19 – Please provide citation for the sentence, 'However, access..... are often low." (line 13-14). Access is about the opportunity to use a service, while engagement is about how a person uses a service after they have gained access. Your sentence (Line 14-15) suggests that

cultural models directly reduce access. However, a cultural belief itself does not change the location of a hospital/clinic or its cost, but it affects a person's decision to use the access that is available. Please clarify. Line 17-19, please provide citation.

Methods:

Page 3, line 55-57 – Suggest rephrasing the research question to improve its readability. Do you mean 'What is the experience of South Asian people with long term conditions regarding the barriers and facilitators to accessing, engaging with, and completing group interventions?

For Figure 1, kindly place the title below the diagram. In the PRISMA flow diagram (top left box), authors should include the results from each database, not just the total (N = 8,348).

Results:

Clearly written.

Discussion:

Generally, not all the themes (e.g., faith and safe space) are being discussed in this section. For example, this paper notes faith as a theme but does not fully unpack its role as both a potential barrier and facilitator. Could authors elaborate on specific contexts or beliefs that lead to these opposing outcomes (with references)? Furthermore, what are the implications of this finding for clinical practice?

I do not see the discussion on how this 'Collaborative relationships helped patients feel pride and empowerment' act as a facilitator in group treatment programs.

Page 23, line 52-56, page 24, line 6-9 and line 22-24 - Suggest including more recent references.

Suggest adding a 'Limitations' section in the discussion.

Lastly, suggest including a PRISMA checklist.

---

| | |
|---|---|
| **Reviewer** | **2** |
| **Name** | **Kabiri, Neda** |
| **Affiliation** | **Tabriz University of Medical Sciences** |
| **Date** | **23-Oct-2025** |
| **COI** | **None** |

---

- It is recommended to replace the "meta-synthesis" with meta-ethnography in your title and all over the manuscript.

- It would be helpful if the reason for choosing meta-ethnography (instead of a systematic review or scoping review) for assessing barriers and facilitators was provided.

- It would be better if a summary of search strategy was provided in the main text.

- Due to the cultural sensitivity of interpretation, it would be helpful if authors provided reflexivity statements.

- It is unclear how low-quality studies affect the analysis, since it was claimed that they weren't excluded.

- Integration of "Lived Experience Advisory Panel" is not clearly described in the manuscript.

- What are the implications for practice?

---

## VERSION 1 - AUTHOR RESPONSE

**Reviewer One**

| No. | Reviewer One Comment | Response |
|---|---|---|
| **1** | Abstract<br>Page 1, line 35-37 – The sentence is incomplete, '….. barriers and facilitators experienced by…' it is not clear barriers and facilitators in what? Please specify the context of these barriers and facilitators. Are they related to accessing healthcare services? | This sentence has been updated to specify the context. |
| **2** | Abstract<br>Page 1, line 48 - The results section mentions about 'cardiovascular disease and diabetes mellitus', but one of keywords include 'Chronic Obstructive Pulmonary Disease (COPD)'. Please clarify this. | Thank you, this has been clarified both within the abstract and results. |
| **3** | Introduction<br>Page 3, line 13-19 – Please provide citation for the sentence, 'However, access….. are often low." (line 13-14). Access is about the opportunity to use a service, while engagement is about how a person uses a service after they have gained access. Your sentence (Line 14- 15) suggests that cultural models directly reduce access. However, a cultural belief itself does not change the location of a hospital/clinic or its cost, but it affects a person's decision to use the access that is available. Please clarify. Line 17-19, please provide citation. | We have added references to support this statement (L.98).<br><br>Thank you, we agree with the point about the difference between access and engagement and have clarified that cultural explanatory models impact help-seeking and engagement, rather than access directly.<br><br>We have also added a reference to support the point that 'Cultural beliefs, particularly beliefs that long-term conditions (LTCs) are fate, can act as a barrier to help-seeking.' |

<table>
<tr><td>4</td><td>Methods<br>Page 3, line 55-57 – Suggest rephrasing the research question to improve its readability. Do you mean ‘What is the experience of South Asian people with long term conditions regarding the barriers and facilitators to accessing, engaging with, and completing group interventions?</td><td>Thank you, we have amended the research question to increase clarity.</td></tr>
<tr><td>5</td><td>Methods<br>For Figure 1, kindly place the title below the diagram.</td><td>The title has been moved below the diagram.</td></tr>
<tr><td>6</td><td>Methods<br>In the PRISMA flow diagram (top left box), authors should include the results from each database, not just the total (N = 8,348).</td><td>All figures from database searches are now included in the PRISMA flow diagram.</td></tr>
<tr><td>7</td><td>Results<br>Clearly written</td><td>Thank you.</td></tr>
<tr><td>8</td><td>Discussion<br>Generally, not all the themes (e.g., faith and safe space) are being discussed in this section. For example, this paper notes faith as a theme but does not fully unpack its role as both a potential barrier and facilitator. Could authors elaborate on specific contexts or beliefs that lead to these opposing outcomes (with references)? Furthermore, what are the implications of this finding for clinical practice?</td><td rowspan="2">Thank you for both of these recommendations relating to the discussion. We have revised the discussion section to address both points. The discussion now expands on the theme of faith, specifically explaining how it can act as both a barrier and facilitator in different contexts, with supporting references. We have also elaborated on how collaborative relationships foster pride and empowerment as facilitators in group treatment programs, which is now more fully integrated into the communications section.</td></tr>
<tr><td>9</td><td>Discussion<br>I do not see the discussion on how this ‘Collaborative relationships helped patients feel pride and empowerment’ act as a facilitator in group treatment programs.</td></tr>
<tr><td>10</td><td>Discussion<br>Suggest adding a ‘Limitations’ section in the discussion.</td><td>Thank you for this comment, this has now been added below the discussion.</td></tr>
<tr><td>11</td><td>Discussion<br>Page 23, line 52-56, page 24, line 6-9 and line 22-24 - Suggest including more recent references.</td><td>Thank you for this comment. While we have retained the citations due to their relevance, we have also added the following recent references to this section:<br>Bhui, K. (2010). Culture, religion and health care.</td></tr>
</table>

| | | |
|---|---|---|
| | | International Journal of Integrated Care, 10(Suppl), e021.<br>Idris, I. B., Hamis, A. A., Bukhori, A. B. M., Hoong, D. C. C., Yusop, H., Shaharuddin, M. A. A., ... & Kandayah, T. (2023). Women's autonomy in healthcare decision making: a systematic review. BMC Women's Health, 23(1), 643.<br>Zota, D., Diamantis, D. V., Katsas, K., Karnaki, P., Tsiampalis, T., Sakowski, P., ... & Linos, A. (2023). Essential skills for health communication, barriers, facilitators and the need for training: Perceptions of healthcare professionals from seven European countries. In Healthcare (Vol. 11, No. 14, p. 2058). MDPI.<br>Bhopal, K. (2019). Gender, 'Race' and patriarchy: a study of South Asian women. Routledge. |
| **12** | Supplementary Materials<br>Lastly, suggest including a PRISMA checklist. | Thank you, we have added a PRISMA checklist in supplementary materials. |

**Reviewer Two**

| No. | Reviewer Two Comment | Response |
|---|---|---|
| **1** | It is recommended to replace the "meta-synthesis" with meta-ethnography in your title and all over the manuscript. | Thank you for highlighting this point. We have removed reference to meta-synthesis and amended to meta-ethnography throughout.<br><br>We have amended our title to reflect that this paper is a systematic review and meta-ethnography. |
| **2** | It would be helpful if the reason for choosing meta-ethnography (instead of a systematic review or | This is a systematic review with meta-ethnography. It applied systematic methods for |

| | | |
|---|---|---|
| | scoping review) for assessing barriers and facilitators was provided. | identifying and appraising qualitative studies and used meta-ethnography as the analytic approach for synthesis.<br><br>Meta-ethnography was chosen as the most appropriate synthesis method because the study aimed to understand how South Asian people with long-term conditions experience and make sense of the factors that influence their access to and engagement with group interventions. This interpretive approach enables a deeper synthesis of qualitative studies, moving beyond simply identifying and listing barriers and facilitators to produce new, novel interpretations whilst preserving the original meanings and study concepts.<br><br>This has been clarified in the title, abstract, introduction, methods, and discussion with appropriate citations. |
| **3** | It would be better if a summary of search strategy was provided in the main text. | Thanks for this comment. The search strategy is now available in both supplementary material and written form (L.176-19):<br>"We conducted systematic searches across six electronic databases from inception to March 2024 (updated April 2025): MEDLINE, PsycINFO, and EMBASE (via Ovid), CINAHL (via EBSCOhost), CENTRAL (via Cochrane Library), and Applied Social Sciences Index and Abstracts (viaProQuest).<br>The search strategy comprised four key components combined using Boolean operators:<br>1. Condition terms for COPD, cardiovascular disease, and diabetes; |

| | | |
|---|---|---|
| | | 2. Intervention terms e.g., self-management, patient education, health promotion, disease management, and rehabilitation programs;<br><br>3. Population terms for South Asian countries (i.e., India, Pakistan, Bangladesh, Sri Lanka, Nepal, Bhutan, Afghanistan, Maldives) and South Asian ethnic groups;<br><br>4. Qualitative research methodology terms e.g., interviews, focus groups, phenomenology, ethnography.<br><br>Search terms were adapted for each database using appropriate MeSH terms and free-text keywords. The full search strategy is provided in supplementary material (S2).” |
| **4** | Due to the cultural sensitivity of interpretation, it would be helpful if authors provided reflexivity statements. | Thank you for highlighting that this was missing from our methods section.<br><br>To address this comment, we have added reflexivity statements where appropriate and in-keeping with reporting according to the eMERGe Guidelines.<br><br>To summarise:<br>- In the methods section ‘Step 2: Deciding What is Relevant’ we have added a reflexivity statement. It highlights the backgrounds of the authors (EVS, JM, AB) who were involved in screening and study selection, and how their professional and lived experiences shaped engagement with the data. It also notes the use of team discussions to reflect on differing |

| | | |
|---|---|---|
| | | assumptions during study screening and interpretation.<br>- In 'Step 6 Synthesising Translations' we have elaborated on reflexivity, describing how the whole authorship team engaged in reflexive discussions, and how we engaged with a lived experience advisory panel and research advisory group. The background of all authors and each group are described. We have also noted that differences in interpretation were explored collaboratively, noted by EVS and incorporated into the line-of-argument synthesis.<br>We have also explained that four authors identify as British South Asian and therefore, provided additional cultural insight into the interpretation of data.<br>- In 'Step 7 Expressing the Synthesis' we have described how the presentation of the synthesis was informed by ongoing reflexive discussions between EVS and AB, with interpretations subsequently sense-checked with the full author team to ensure the synthesis accurately reflected the data and considered multiple perspectives, including health-care professional, researcher, community engagement, and lived-experience. |

| | | |
|---|---|---|
| **5** | It is unclear how low-quality studies affect the analysis, since it was claimed that they weren't excluded. | Thank you for the opportunity to clarify this. Low-quality studies were not excluded. However, during the reciprocal translation phase of our meta-ethnography articles were ranked by quality from highest to lowest. This ranking informed the interpretive weighting of each study during our synthesis. This is outlined in our methods section 'Step 5: Translating the Studies Into One Another.,' which we have expanded to ensure that our approach to the quality of included papers within the synthesis is clear. |
| **6** | Integration of "Lived Experience Advisory Panel" is not clearly described in the manuscript. | Thank you for this comment. As detailed in our response to Editor Comment 2 (Patient and Public Involvement statement), we have clarified the use of our LEAP in two ways:<br>1. Added early in the methods to highlight who the LEAP was and when they were involved (L.130-137);<br>2. Revised Step 6 and 7 to specify the use of the LEAP throughout translation and synthesis.<br>We feel these changes make it clearer that the LEAP provided ongoing input rather than a single consultation. |
| **7** | What are the implications for practice? | We appreciate this comment. Implications for practice are highlighted throughout the discussion. Key recommendations for clinicians and policymakers include culturally sensitive assessments considering faith, gender-sensitive approaches, improved communication strategies, longer appointments for patients with limited English proficiency, |

| | | |
|---|---|---|
| | | translated materials, increased workforce diversity, and engagement with community leaders. These points illustrate how our findings can inform actionable strategies to enhance engagement with group health interventions among South Asian patients. |
